# Danlian-Tongmai formula improves diabetic vascular calcification by regulating CCN3/NOTCH signal axis to inhibit inflammatory reaction

**DOI:** 10.3389/fphar.2024.1510030

**Published:** 2025-01-06

**Authors:** Wenting Wang, Yiwen Li, Mengmeng Zhu, Qian Xu, Jing Cui, Yanfei Liu, Yue Liu

**Affiliations:** ^1^ National Clinical Research Center for TCM Cardiology, Xiyuan Hospital, China Academy of Chinese Medical Sciences, Beijing, China; ^2^ Beijing Key Laboratory of Traditional Chinese Medicine Basic Research on Prevention and Treatment for Major Diseases, Experimental Research Center, China Academy of Chinese Medical Sciences, Beijing, China; ^3^ The Second Department of Geriatrics, Xiyuan Hospital, China Academy of Chinese Medical Sciences, Beijing, China

**Keywords:** vascular calcification, Danlian-Tongmai formula, UHPLC-MS, CCN3, notch signaling

## Abstract

**Background:**

Vascular calcification (VC) commonly occurs in diabetes and is associated with cardiovascular disease incidence and mortality. Currently, there is no drug treatment for VC. The Danlian-Tongmai formula (DLTM) is a traditional Chinese medicine (TCM) prescription used for diabetic VC (DVC), but its mechanisms of action remain unclear. This study aims to elucidate the effects of DLTM on DVC and explore the underlying mechanisms of action.

**Methods:**

Ultra-high-performance liquid chromatography-mass spectrometry (UHPLC-MS) was used to identify the metabolites of DLTM. A DVC rat model was established using streptozotocin (STZ) combined with vitamin D3 (VitD3). The effects of DLTM on DVC were evaluated through alizarin red staining, calcium deposition, and changes in osteogenic and contractile markers. The specific molecular mechanism of DLTM in treating diabetic VC was comprehensively analyzed by transcriptomics, molecular docking and *in vivo* experimental verification.

**Results:**

We identified 108 major metabolites of DLTM. *In vivo*, high-dose DLTM significantly alleviated VC in diabetic rats. Transcriptomic analysis showed that DLTM treatment markedly altered the transcriptomic profile of rat aortas, which was associated with regulating the CCN3/NOTCH signaling pathway, promoting vascular smooth muscle contraction, and inhibiting the inflammatory responses. Molecular docking and molecular dynamics simulation demonstrated strong binding interactions between DLTM metabolites and key molecules within the CCN3/NOTCH pathway, including NOTCH1, DLL1, DLL4, hes1, and hey1. *In vivo* experiments confirmed that DLTM could upregulate CCN3, inhibit the activation of NOTCH signaling ligands DLL1 and downstream transcription factors hes1 and hey1, and reduce the release of inflammatory cytokines IL6, IL1β, and TNFα.

**Conclusion:**

DLTM alleviates DVC by regulating the CCN3/NOTCH signaling axis to inhibit inflammatory responses. Our research provides experimental basis for clinical treatment and drug transformation of diabetic VC.

## 1 Introduction

Vascular calcification (VC) is a pathological vascular disorder characterized by the ectopic deposition of calcium phosphate in the form of hydroxyapatite within the vascular wall. It is highly prevalent in diabetic patients and is the pathological basis for macrovascular complications in diabetes (Lee et al., 2020; Yao et al., 2021). The increase in VC at different sites can lead to the rupture of vulnerable plaques, vascular stiffness, and loss of elastic buffering, resulting in unstable hemodynamic consequences (Vengrenyuk et al., 2006). Diabetic patients with severe VC have a tenfold higher risk of cardiovascular events compared with those without VC (Wong and Sattar, 2023). In addition, the progression and severity of VC are highly correlated with adverse cardiovascular events such as acute coronary syndromes and sudden cardiac death, making VC a well-recognized independent predictor of all-cause mortality and lifetime cardiovascular disease risk in diabetic patients (Lei et al., 2021; Ferket et al., 2022).

Previously considered a passive, degenerative vascular condition, VC is now recognized as an active, multi-gene regulated biological process, similar to osteogenic differentiation in skeletal mineralization, primarily driven by vascular smooth muscle cells (VSMCs) (Quaglino et al., 2020). Systemic metabolic disorders caused by type 2 diabetes mellitus (T2DM), including chronic hyperglycemia, dyslipidemia, and insulin resistance, are key factors promoting the development of VC (Yahagi et al., 2017). Endothelial dysfunction is the initiating factor in diabetic VC (DVC) (Jiang et al., 2022). Hyperglycemia stimulates the progression of VC from the endothelial cells gradually to the entire vascular media and adventitia, accompanied by continuous deposition of calcium and phosphate ions, exacerbation of local vascular oxidative stress and inflammatory responses, and imbalance in the expression of factors associated with bone homeostasis. These processes promote the differentiation of VSMCs from a contractile phenotype to an osteogenic phenotype, thereby inducing the ectopic growth of hydroxyapatite within the vasculature and ultimately leading to VC. VC is a common vascular pathology in diabetic patients, with an increasingly younger age of onset. However, the exact pathogenic mechanisms remain unclear, and effective preventive and therapeutic drugs are lacking. Actively exploring strategies for the prevention and treatment of VC is crucial for improving symptoms in diabetic patients and reducing the risk of cardiovascular mortality.

Traditional Chinese medicine (TCM) has demonstrated excellent efficacy and significant potential in preventing and treating diabetes and its complications owing to its multi-metabolite, multi-target, and multi-pathway characteristics (Tian et al., 2019). Some clinical studies found that certain TCM formulas, which enhance qi and promote blood circulation, can reduce VC by alleviating glucolipotoxicity, inhibiting VSMC apoptosis, and preventing osteogenic transformation (Yang et al., 2023). Additionally, specific TCM monomers, such as resveratrol and astragaloside IV, have been found to effectively inhibit VSMC proliferation, migration, autophagy, and phenotypic transformation *in vitro* (Song et al., 2019; Huang et al., 2022). These findings suggest that TCM and its natural metabolites can serve as potential therapeutic drugs for DVC. According to TCM theory, the pathogenesis of DVC can be attributed to blood stasis obstructing the meridians, toxic stasis accumulation, and deficiency of Qi. The treatment approach involves promoting blood circulation to remove stasis, detoxifying, and replenishing Qi while unblocking the meridians. Based on this understanding, Xiyuan Hospital of the China Academy of Chinese Medical Sciences developed the Danlian-Tongmai formula (DLTM), a specialized prescription for VC. It consists of five botanical drugs, namely *Salvia miltiorrhiza* Bunge., *Coptis chinensis* Franch., *Polygonum cuspidatum* Siebold & Zucc., *Prunus mume* (Siebold) Siebold & Zucc., and *Rhodiola crenulata* (Hook.f. & Thomson) H. Ohba, and has been granted a patent for its invention (Patent number: 202311796613.6). Among them, *Salvia miltiorrhiza* Bunge. has the effects of promoting blood circulation, removing blood stasis and dredging veins, and is widely used in the clinical treatment of diabetic vascular complications (MEIm et al., 2019). Its active metabolite, Tanshinone II, can inhibit the activation of inflammatory signals such as NF-κB, thereby preventing the osteogenic transformation of VSMCs, demonstrating pharmacological effects in the prevention of VC (Zhong et al., 2022). *Coptis chinensis* Franch. is a common traditional Chinese medicine with significant heat-removing and detoxifying activities, It has pharmacological effects such as lowering blood sugar, anti-bacterial, antioxidant, and anti-inflammatory properties (Wang et al., 2019). Clinically, it is often used to treat diabetic atherosclerosis, diabetic nephropathy, and other diabetic vascular complications (Jin et al., 2020; Zhang and Zheng, 2024). It has been reported that the active metabolite of Coptis chinensis, berberine, can improve high glucose-induced VSMC calcification and VC by inhibiting endoplasmic reticulum stress and apoptosis-related pathways (Li et al., 2022). *Polygonum cuspidatum* Siebold & Zucc. is a medicinal plant with a long history, known for its heat-clearing, detoxifying, blood-activating, and stasis-dissolving effects. Numerous studies have confirmed that extracts of *Polygonum cuspidatum* Siebold & Zucc. and its active metabolites can effectively alleviate vascular complications such as mesangial cell dysfunction and retinopathy caused by diabetes (Sohn et al., 2014; Sohn et al., 2016), and can inhibit the osteogenic transformation of VSMCs induced by high glucose and vascular sclerosis caused by a high-fat and high-glucose diet (Mattison et al., 2014; Zhang et al., 2016). *Prunus mume* (Siebold) Siebold & Zucc. is rich in organic acids and flavonoids, is commonly used in traditional Chinese medicine for the treatment of diabetes (Li et al., 2013; Tu et al., 2013). Studies have shown that *Prunus mume* (Siebold) Siebold & Zucc. extract participates in regulating osteogenic differentiation of cells and can reduce vascular remodeling through its anti-inflammatory properties (Kono et al., 2011; Okuno et al., 2023). *Rhodiola crenulata* (Hook.f. & Thomson) H. Ohba. has the effects of tonifying qi, nourishing blood, and relieving asthma, and it is widely used in the treatment of cancer, diabetes, oxidative damage, and neuroprotection (Bassa et al., 2016; Déciga-Campos et al., 2016; Xu et al., 2018). Its main metabolite, salidroside, significantly inhibits high glucose-induced VSMC migration and osteogenic phenotypic transformation, restoring the contractile phenotype of VSMCs(Zhang et al., 2020). Despite the pharmacological evidence and significant clinical efficacy, the efficacy and mechanism of DLTM in treating diabetic VC are still unclear due to its complex metabolites. This study aims to clarify the therapeutic effects of DLTM on DVC using a rat model induced by streptozotocin (STZ) combined with vitamin D3 (VitD_3_). Additionally, high-throughput transcriptomics, molecular docking, and *in vivo* experiments were conducted to systematically explore the pharmacological mechanisms of DLTM. This research aims to provide experimental evidence for a deeper understanding of the pathogenesis of DVC and support the clinical translation of DLTM.

## 2 Materials and methods

### 2.1 Animal model

Six-week-old male Sprague Dawley (SD) rats were purchased from SiPeiFu (Beijing) Biotechnology Co., Ltd. (SCXK (Beijing) 2019–0010). The rats were housed in cages at a temperature of 22°C ± 2°C and humidity of 55% ± 5%, with a 12/12-h light/dark cycle in an SPF animal facility (containing sterilized water and feed). The animal experiment was approved by the Animal Ethics Committee of Xiyuan Hospital of China Academy of Chinese Medical Sciences (approval number: 2024XLC014-2) and adhered to the Guide for the Care and Use of Laboratory Animals from the National Institute of Health (NIH Publication No. 85–23, revised 1996).

We employed an STZ combined with VitD3 overload to induce the DVC model. STZ is a β-cell toxin and is the most commonly used method to induce diabetes in rodent models (Furman, 2021), and it is widely applied in the study of diabetic vascular complications (Stabley and Towler, 2017). STZ not only destroys pancreatic β-cells but also accelerates vascular injury and biomineralization reactions through DNA alkylation (Bennett and Pegg, 1981). This results in aggravated vascular endothelial cells dysfunction (Mao et al., 2024), along with increased mineral deposition and stiffness (Heath et al., 2014), which is more closely mimic the clinical conditions observed in patients with diabetic vascular complications. After 1 week of acclimatization, intraperitoneal injections of STZ (Sigma-Aldrich, United States) at a dose of 30 mg/kg were administered to the rats for three consecutive days. One week later, fasting blood glucose levels were measured. Rats with blood glucose levels ≥11.1 mmol/L and pronounced symptoms of “polyuria, polydipsia, and weight loss” were considered to have successfully developed diabetes to accelerate VC development and mitigate the effects of aging on VC formation, a previously reported method (Yang et al., 2023) was adopted and improved by using VitD_3_ (Sigma-Aldrich, United States) overload to induce DVC. Specifically, diabetic rats received intraperitoneal injections of high-dose VitD_3_ (5.5 × 10^5^ IU/kg) for four consecutive days. 14 days after the first injection is considered as the end of calcification modeling period, and its modeling success rate has been proved to be over 80% (Lu et al., 2024). Another group of SD rats were used as a normal control group (CON group) and received equivalent volumes of vehicle (pure water) instead of the STZ and VitD_3_ injections. All groups of rats were granted free access to food and water throughout the study period.

### 2.2 Preparation and quality control of DLTM

The DLTM comprises of *Salvia miltiorrhiza* Bunge. (20 g), *Coptis chinensis* Franch. (10 g), *Polygonum cuspidatum* Siebold & Zucc. (10 g), *Prunus mume* (Siebold) Siebold & Zucc. (10 g), and *Rhodiola crenulata* (Hook.f. & Thomson) H. Ohba. (10 g), and mixed at a ratio of 2:1:1:1:1. In brief, 600 g of *Salvia miltiorrhiza* Bunge., 300 g of *Coptis chinensis* Franch., 300 g of *Polygonum cuspidatum* Siebold & Zucc., 300 g of *Rhodiola crenulata* (Hook.f. & Thomson) H. Ohba., and 300 g of *Prunus mume* (Siebold) Siebold & Zucc. were weighed. These botanical drugs were provided by Institute of Traditional Chinese Medicine, Chinese Academy of Traditional Chinese Medicine. The botanical drugs decocted twice with eight and six times the amount of pure water, respectively, for 1 h each time. The decoctions were subsequently filtered, combined, and left to stand overnight, after which the supernatant was collected. The supernatant was passed through a 6L SP825 macroporous resin column at a flow rate of 4.2 L/h, washed with 4 bed volumes (BV) of water, dried, and eluted with 3 BV of 65% ethanol. The ethanol was then recovered, and the solution was concentrated into a thick paste. This process was repeated, and the pastes were combined, vacuum-dried at 80°C, ground into a fine powder, and mixed with dextrin to a final weight of 153.2 g. Each gram of dry extract contained 23.5 g of crude drug.

### 2.3 Drug administration

After model induction, the DVC rats were randomly divided into a model (DVC group), low-dose DLTM (DLTM-L group), medium-dose DLTM (DLTM-M group), high-dose DLTM (DLTM-H group), or positive control Dapagliflozin (DAPA group) group (n = 12 for each group). The groups received different doses of DLTM and Dapagliflozin (AstraZeneca Pharmaceuticals, United States) by gavage for 4 weeks. The dosage of DLTM is determined with reference to the clinically equivalent dose, and the dose gradients are set at 1, 2, and 4 times the equivalent dose based on previous studies (Liu et al., 2024). Specifically, The human equivalent oral dose of DLTM dry extract (0.04 g/kg/day for a 60 kg person) was converted to the rat equivalent dose based on body surface area (BSA) normalization (Reagan-Shaw et al., 2008), resulting in doses of 0.24 g/kg dry extract for the DLTM-L group, 0.48 g/kg dry extract for the DLTM-M group, and 0.96 g/kg dry extract for the DLTM-H group. Alternatively, the DAPA group received oral Dapagliflozin at 5 mg/kg/day based on a previous study (Zhang et al., 2021), while the DVC and CON groups received equivalent volumes of the vehicle by gavage. The survival status, body weight, and fasting blood glucose of the rats were recorded weekly. After the drug intervention period, the rats were fasted for 12 h, anesthetized with an intraperitoneal injection of sodium pentobarbital (30 mg/kg), and blood and aortic samples were collected for further analysis.

### 2.4 Ultra-high-performance liquid chromatography-mass spectrometry (UHPLC-MS)

UHPLC-MS was conducted using a Vanquish UHPLC system (Thermo Fisher Scientific, United States) coupled with a Q Exactive HF-X mass spectrometer (Thermo Fisher Scientific, United States). The chromatographic column used was a HYPERSIL GOLD C18 (100 × 2.1 mm, 1.9 μm, Thermo Fisher Scientific, United States). The flow rate was set to 0.3 mL min^−1^, the column temperature to 40°C, and the injection volume to 5 μL. The mobile phases consisted of 0.1% formic acid in water (solvent A) and 0.1% formic acid in acetonitrile (solvent B). The gradient elution program was as follows: 0–3.0 min, 5% B; 3.0–8.0 min, 5%–14% B; 8.0–16.0 min, 14%–40% B; 16.0–21.0 min, 40%–45% B; 21.0–26.0 min, 45%–65% B; 26.0–29.0 min, 65%–100% B; 29.0–34.0 min, 100% B; 34.0–35.0 min, 100%–5% B; and 35.0–40.0 min, 5% B.

Metabolite analysis was performed using an electrospray ionization (ESI) source with the following parameters: sheath gas flow rate, 40 arb; auxiliary gas flow rate, 10 arb; ion transfer tube temperature, 320°C; auxiliary gas heater temperature, 350°C; S-Lens RF level, 60 V; full MS resolution, 120,000; MS/MS resolution, 60,000; and scan range, 100–1,500 m/z. The spray voltage was 3.8 kV for positive ion mode and 3.0 kV for negative ion mode.

### 2.5 Alizarin red S staining

As previously described (Lu et al., 2024), alizarin red staining was employed to assess arterial calcification. For whole aorta staining, the aorta was fixed in 95% ethanol for 24 h and stained overnight with 1% alizarin red solution (Beyotime, C0140). The arterial tissue was then rinsed with 2% potassium hydroxide and photographed using an inverted microscope.

For aortic ring staining, freshly isolated aortic arches were fixed in 4% paraformaldehyde, embedded in paraffin, sectioned at a thickness of 5 μm, and deparaffinized. The aortic sections were subsequently stained with 1% alizarin red solution for 5 min and photographed using an inverted microscope. Quantitative analysis of positively stained areas was performed using Image J software.

### 2.6 Alkaline phosphatase (ALP) activity and calcium content detection

Aortic tissues were homogenized and centrifuged to separate the supernatant. ALP activity in the supernatant was then measured using an ALP assay kit (Beyotime, P0321S). Calcium content was determined using a commercial calcium assay kit (Beyotime, S1063S) according to the manufacturer’s instructions. Briefly, the supernatant was mixed with methylthymol blue (MTB), an alkaline solution, and a protein clearing reagent, and incubated at room temperature for 5 min. The absorbance was measured at 610 nm using a microplate reader, and the relative calcium content was normalized to the protein content.

### 2.7 Serum biochemical analysis

Fasting blood glucose levels were measured weekly using a portable glucometer (ROCHE, Germany) after STZ injections. Serum calcium and phosphorus levels were measured using a Mindray BS-420 automatic biochemical analyzer (Mindray, China).

### 2.8 Enzyme-linked immunosorbent assay

Serum levels of IL-6, IL-1β, and TNF-α were measured using enzyme-linked immunosorbent assay (ELISA) kits (MEIMIAN, China) with catalog numbers MM-0190R2, MM-0047R2, and MM-70625R2, respectively. The assay procedures adhered to the manufacturer’s instructions.

### 2.9 Transcriptome RNA sequencing

Total RNA was extracted from the rat aorta using Trizol reagent (CapitalBio Technology, Beijing) following the manufacturer’s protocol. RNA integrity was assessed using an Agilent 2,100 Bioanalyzer (Agilent Technologies, United States). Libraries were prepared using the TruSeq Stranded Total RNA and Ribo-Zero Gold kits (Illumina) and sequenced on an Illumina NovaSeq 6,000 platform (Illumina, United States). Raw data in fastq format were filtered using fastp software, and clean reads were mapped to the rat genome (Rnor_6.0 Ensembl 104) using Hisat2. Gene and transcript expression levels were quantified using featureCounts and StringTie. Differential expression analysis was performed using the limma R package, with thresholds set at *p* < 0.05 and |log2 (Fold Change)| ≥ 1.2 for significant differentially expressed genes (DEGs). DEG enrichment analysis was conducted using DAVID 6.8 and further analyzed using R packages.

### 2.10 Quantitative real-time polymerase chain reaction (qPCR)

Total RNA was extracted from the aorta using Trizol reagent (Invitrogen, United States) according to the manufacturer’s instructions, the concentration of which was measured using a NanoDrop spectrophotometer (Thermo Fisher Scientific, United States). RNA was reverse-transcribed into complementary DNA (cDNA) using the M-MLV Reverse Transcriptase kit (Promega, United States). PCR amplification was performed on the obtained cDNA using the QuantiTect Multiplex RT-PCR Kit (Qiagen, Germany). Real-time qPCR was conducted using SYBR Green Master Mix (Takara, Japan) on a 7500 FAST Real-Time PCR System (Applied Biosystems, United States). With β-actin as the internal control, relative mRNA expression levels were standardized and analyzed using the 2^−ΔΔCT^ method. Primer sequences specific to target genes are listed in Table 1.

**TABLE 1:** Primers for qPCR.

Primer	Sequence (5′to 3′)	Sizes of PCR products
Ccn3-F	TGG​TTC​CAG​AGG​GAG​ACA​AC	114 bp
Ccn3-R	CAC​AGC​CAA​TTT​GCC​CAT​CT	
Sfrp4-F	GAG​GAG​CTG​GTA​GAC​GTG​AA	177 bp
Sfrp4-R	TGG​CCA​GCT​GTG​GTT​ATA​CA	
Tpm2-F	AGA​AGC​TGA​AGG​AGG​CTG​AG	105 bp
Tpm2-R	CCT​TGG​CAC​TAG​CCA​AAG​TC	
β-actin-F	GGC​TGA​TTC​CCC​TCC​ATC​G	154 bp
β-actin-R	CCA​GTT​GGT​AAC​AAT​GCC​ATG​T	

Note: F (Forward); R (Reverse).

### 2.11 Western blot analysis

Aortic tissues were lysed in RIPA buffer containing protease and phosphatase inhibitors. Protein concentration subsequently was determined using a BCA protein assay kit (Solarbio, China). Next, proteins were separated by 10% sodium dodecyl sulfate-polyacrylamide gel electrophoresis (SDS-PAGE) and transferred onto polyvinylidene fluoride (PVDF) membranes (Millipore, United States). These membranes were blocked with 5% non-fat milk for 1 h at room temperature and incubated with primary antibodies overnight at 4°C. The next day, the membranes were further incubated with HRP-conjugated secondary antibodies for 1 h at room temperature. Bands were visualized using an imaging system (Amersham Imager 600) and quantified with ImageJ software. The primary antibodies used in Western blot are listed in Supplementary Table S1.

### 2.12 Molecular docking and molecular dynamics simulation

The acquired DLTM metabolites were screened based on Lipinski’s rule of five followed by using SwissADME (http://www.swissadme.ch/) to identify those with high gastrointestinal (GI) absorption and at least three out of five Lipinski, Ghose, Veber, Egan, and Muegge criteria (Lipinski et al., 2001). A total of 59 metabolites were identified for molecular docking. The protein 3D structures of these metabolites were obtained from the PDB database (http://www.pdb.org/) and saved as PDB files. The only exception was DLL3, whose structure was not available in the PDB dataset and was therefore predicted using the AlphaFold Protein Structure Database. Subsequently, PyMOL 2.4.1 was employed to remove water and ligands from proteins, AutoDock Vina 1.1.2 for hydrogen addition and charge calculation, and ChemOffice 22 for minimizing the energy of small molecule metabolites. Molecular docking was performed using AutoDock Vina 1.1.2. The binding affinity between targets and metabolites was evaluated using the docking score.

The protein-ligand complexes with the lowest binding energy was subjected to a 100 ns molecular dynamics simulation using Gromacs 2023 (Abraham et al., 2015). The topology of the protein was constructed using the CHARMM36 (Jo et al., 2008) force field parameters, while the topology of the ligand was generated based on the GAFF2 force field parameters. The protein-ligand complex was placed within a cubic box, the TIP3P water model was then used to fill the box with water molecules (Harrach and Drossel, 2014). Electrostatic interactions were handled using the Particle Mesh Ewald (PME) method and the Verlet algorithm. Subsequently, the system underwent 100,000 steps of isothermal-isobaric ensemble equilibration and isothermal-isochoric ensemble equilibration, with a coupling constant of 0.1 picoseconds, lasting for a duration of 100 picoseconds. Both van der Waals and Coulomb interactions were calculated using a cutoff distance of 1.0 nm. Finally, the system was subjected to molecular dynamics simulation under constant temperature (300 K) and constant pressure (1 bar) using Gromacs 2023, totaling 5,000,000 steps with a step size of 2 femtoseconds, resulting in a total simulation time of 100 nanoseconds. Using GROMACS internal tools to evaluate root-mean-square deviation (RMSD), radius of gyration (Rg), solvent accessible surface area (SASA), root-mean-square fluctuation (RMSF) and hydrogen bond formation (HBond). We used MM/PBSA to calculate the binding free energies of all complexes.

### 2.13 Statistical analysis

Statistical analysis and data visualization were conducted using IBM SPSS Statistics (V26.0), GraphPad Prism (V9.5.0), and ImageJ software. Normally distributed continuous variables are presented as mean ± SEM, while non-normally distributed variables are presented as median and interquartile range. For normally distributed data, one-way ANOVA or t-tests were used to determine statistical significance of differences among groups. For non-normally distributed data, the Kruskal–Wallis or Mann-Whitney U test was instead utilized. A *p*-value <0.05 was considered statistically significant.

## 3 Results

### 3.1 UHPLC-MS analysis of DLTM

**FIGURE 1:**
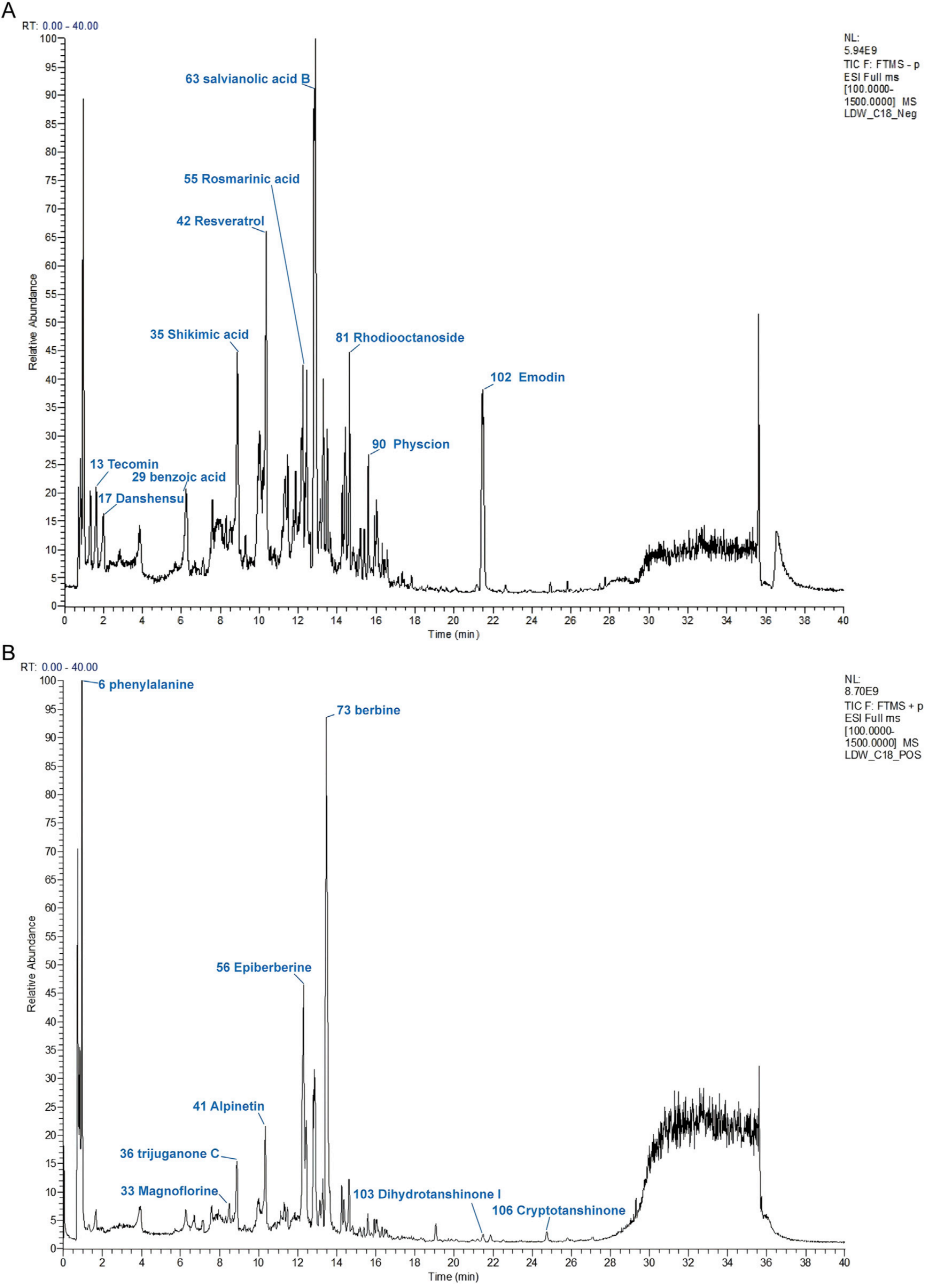
The chromatographic fingerprint of DLTM. **(A)** Ion flow diagram of DLTM in negative ion mode. **(B)** Ion flow diagram of DLTM in positive ion mode. Molecular weight and formula of these metabolites are listed in Supplementary Table S2.

UPLC-MS/MS was employed to establish the quality control of DLTM and to quantify its key metabolites. A total of 108 metabolites were identified (Figure 1), derived from the five botanical drugs, namely *Salvia miltiorrhiza* Bunge., *Coptis chinensis* Franch., *Polygonum cuspidatum* Siebold & Zucc., *Prunus mume* (Siebold) Siebold & Zucc., and *Rhodiola crenulata* (Hook.f. & Thomson) H. Ohba. Specifically, 28 metabolites were identified from *Salvia miltiorrhiza* Bunge., 28 from *Coptis chinensis* Franch., 21 from *Polygonum cuspidatum* Siebold & Zucc., 25 from *Prunus mume* (Siebold) Siebold & Zucc., and 27 from *Rhodiola crenulata* (Hook.f. & Thomson) H. Ohba. Notably, some metabolites were present in multiple botanical drugs. For instance, ferulic acid was found in all five botanical drugs. Detailed information on all identified metabolites in DLTM is provided in Supplementary Table S2.

### 3.2 DLTM significantly improves VC in VitD_3_-Induced DVC rats

**FIGURE 2:**
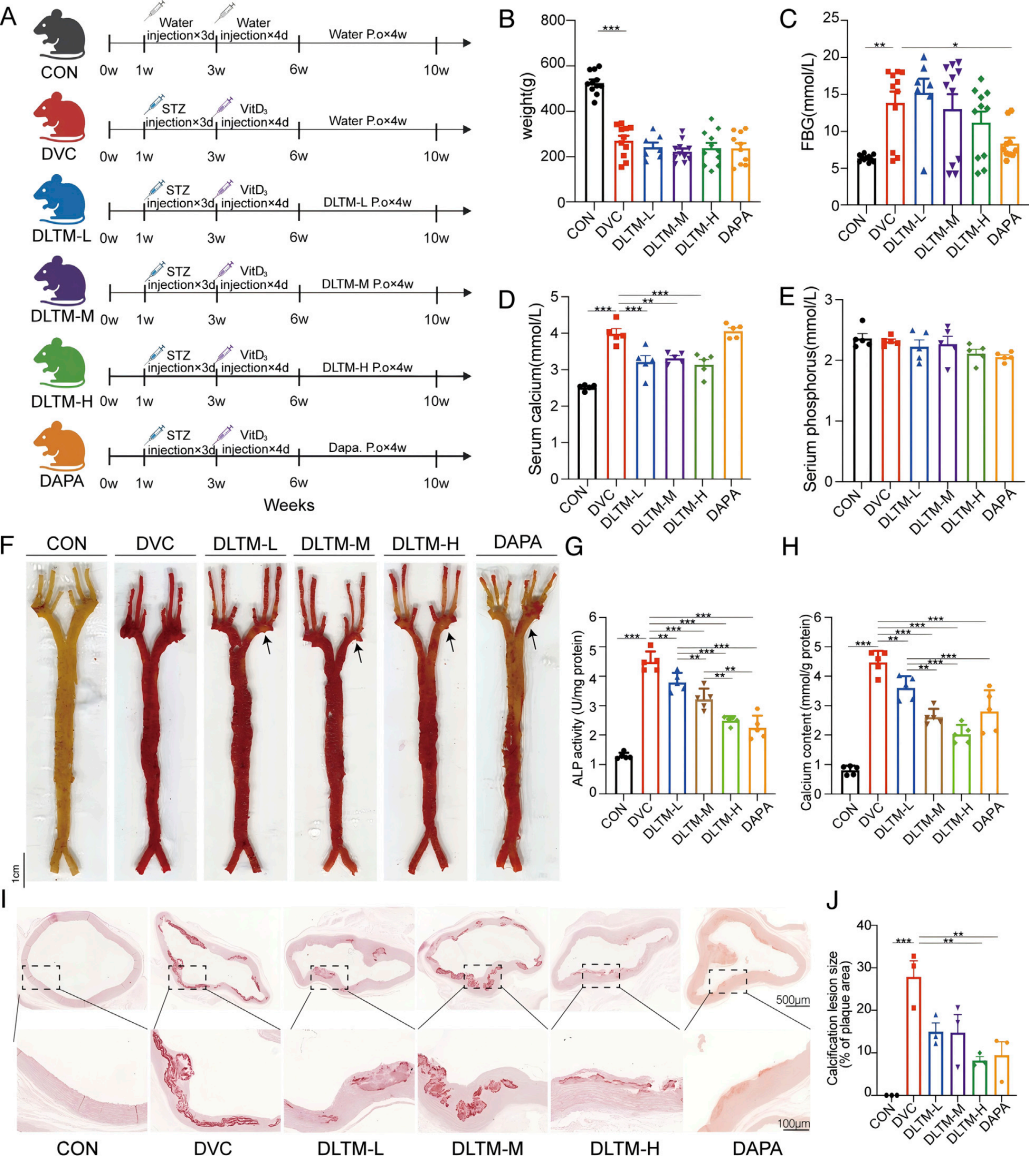
Ameliorative effect of DLTM on vascular calcification in DVC rat. **(A)** Schematic diagram of the experimental design for the rat model; **(B)** Body weight of rats after receiving VitD_3_+STZ or vehicle injection and oral administration of different doses of DLTM, Dapagliflozin, or vehicle for 4 weeks (n = 7–11); **(C)** Fasting blood glucose levels of rats after receiving VitD_3_+STZ or vehicle injection and oral administration of different doses of DLTM, Dapagliflozin, or vehicle for 4 weeks (n = 7–11); **(D)** Serum calcium levels in each group of rats (n = 5); **(E)** Serum phosphorus levels in each group of rats (n = 5); **(F)** Representative images of whole aorta alizarin red staining in each group of rats (n = 3); **(G)** Vascular ALP levels of each group of rats (n = 5); **(H)**. Vascular calcium content of each group of rats (n = 5); **(I)** Representative images of alizarin red-stained aortic arch sections in each group of rats (n = 3,4 × 20×); **(J)** Quantitative analysis of the alizarin red positive staining area in aortic sections from I (n = 3). *P < 0.05, **P < 0.01, ***P < 0.001.

To elucidate the effect of DLTM on DVC, a DVC rat model was established using STZ combined with VitD_3_, with Dapagliflozin as a positive control. Previous studies have confirmed the efficacy of SGLT2 inhibitors in treating DVC (Chen et al., 2023; Li X. et al., 2024). For example, Dapagliflozin has been shown to significantly alleviate arterial calcification by suppressing endoplasmic reticulum stress and the osteogenic transdifferentiation of VSMCs (Li L. et al., 2024; Wu et al., 2024). In this study, after 4 weeks of DLTM or Dapagliflozin administration, the overall condition and vascular damage of the rats were assessed (Figure 2A). As shown, the DVC group exhibited typical diabetic symptoms such as emaciation and elevated blood glucose compared to the CON group (Figures 2B, C; Supplementary Figure S1; Supplementary Table S3, *P* < 0.05). VitD_3_ injection significantly increased serum calcium levels in rats (Figure 2D; Supplementary Table S3, *P* < 0.05) but exhibited no effect on serum phosphorus levels (Figure 2E; Supplementary Table S3, *P* > 0.05). Compared to the DVC group, different doses of DLTM effectively reduced serum calcium levels in rats (Figure 2D, *P* < 0.05, *P* < 0.01), but there was no significant improvement in body weight, FBG and serum phosphorus levels (Figures 2B, C, E, *P* > 0.05). In contrast, Dapagliflozin effectively lowered blood glucose levels in rats (Figure 2C, *P* < 0.05) but had no significant effect on body weight and calcium-phosphorus metabolism (Figures 2B, D, E, *P* > 0.05). Regarding vascular injury, Gross and aortic alizarin red staining revealed extensive calcium deposition in the aortas of DVC rats (Figures 2F, I, J, *P* < 0.05), consistent with significant increases observed in vascular calcium content and ALP levels (Figures 2G, H, *P* < 0.001). Alizarin red staining showed a dose-dependent reduction in aortic calcium deposition in DVC rats treated with DLTM, with high-dose DLTM significantly outperformed low and medium doses of DLTM in improving aortic calcification in rats, reducing vascular calcium content and ALP activity, and exhibited effects comparable to those of Dapagliflozin (Figures 2F–J, *P* < 0.05). These results collectively indicate that high-dose DLTM and Dapagliflozin effectively inhibit VitD3-induced VC in diabetic rats, with DLTM offering vascular protection independent of glucose lowering.

### 3.3 DLTM effectively alleviates the osteogenic differentiation of VSMCs

**FIGURE 3:**
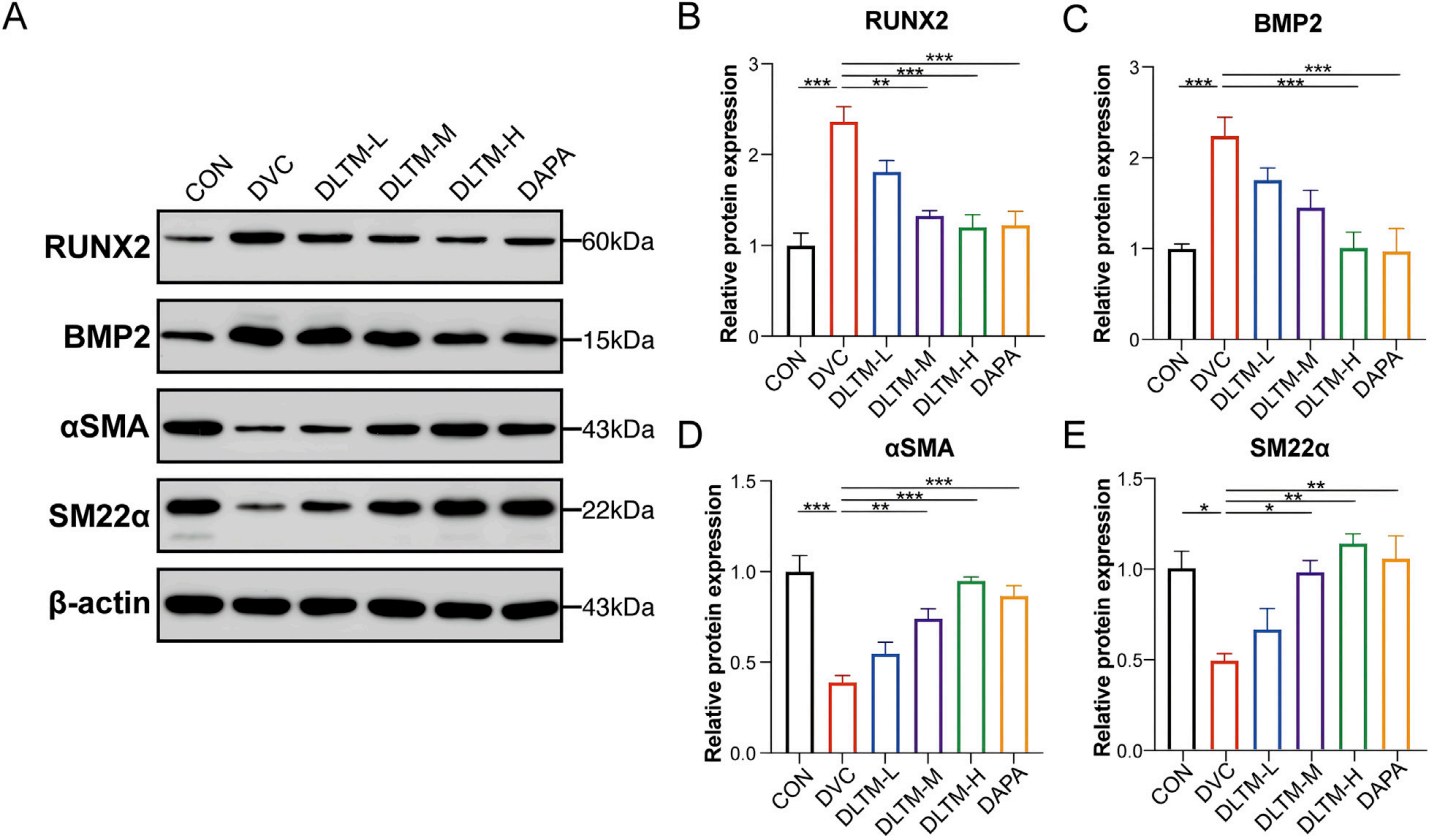
DLTM alleviated osteogenic differentiation of VSMC *in vivo*. **(A)** Representative Western blot images of RUNX2, BMP2, αSMA, and SM22α expression in the aortae of rats from each group; **(B)** Quantitative analysis of RUNX2 expression in **(A, C)** Quantitative analysis of BMP2 expression in **(A, D)** Quantitative analysis of αSMA expression in **(A, E)** Quantitative analysis of SM22α expression in **(A)**. ^*^ *P* < 0.05, ^**^ *P* < 0.01, ^***^ *P* < 0.001.

VSMCs are the primary sites of VC. Their transformation from a contractile to an osteogenic phenotype is a hallmark of DVC, characterized by upregulation of osteogenic proteins such as RUNX2 and downregulation of contractile proteins such α-SMA (Lee et al., 2020). In this study, the extent of VSMC transdifferentiation in the rat aorta was assessed. The results showed that, compared to the CON group, STZ combined with VitD_3_ injection significantly increased the expressions of BMP2 and RUNX2 in the rat aorta while decreasing the expressions of contractile proteins α-SMA and SM22α (Figures 3A–E, *P* < 0.001, *P* < 0.05). In contrast, oral administration of high-dose DLTM or Dapagliflozin effectively reversed the upregulation of osteogenic markers RUNX2 and BMP2, while increasing α-SMA and SM22α levels in the aorta of DVC rats (Figures 3A–E, *P* < 0.001, *P* < 0.01, *P* < 0.05). In contrast, the effects of medium-dose and low-dose DLTM on these osteogenic and contraction markers were limited, with some markers showing no significant improvement (Figures 3A–E, *P>* 0.05). In summary, these findings suggest that DLTM can mitigate the osteogenic transformation of VSMCs *in vivo*, thereby preventing further progression of VC.

### 3.4 DLTM exerts Anti-VC effects by modulating inflammation and vascular smooth muscle contraction

**FIGURE 4:**
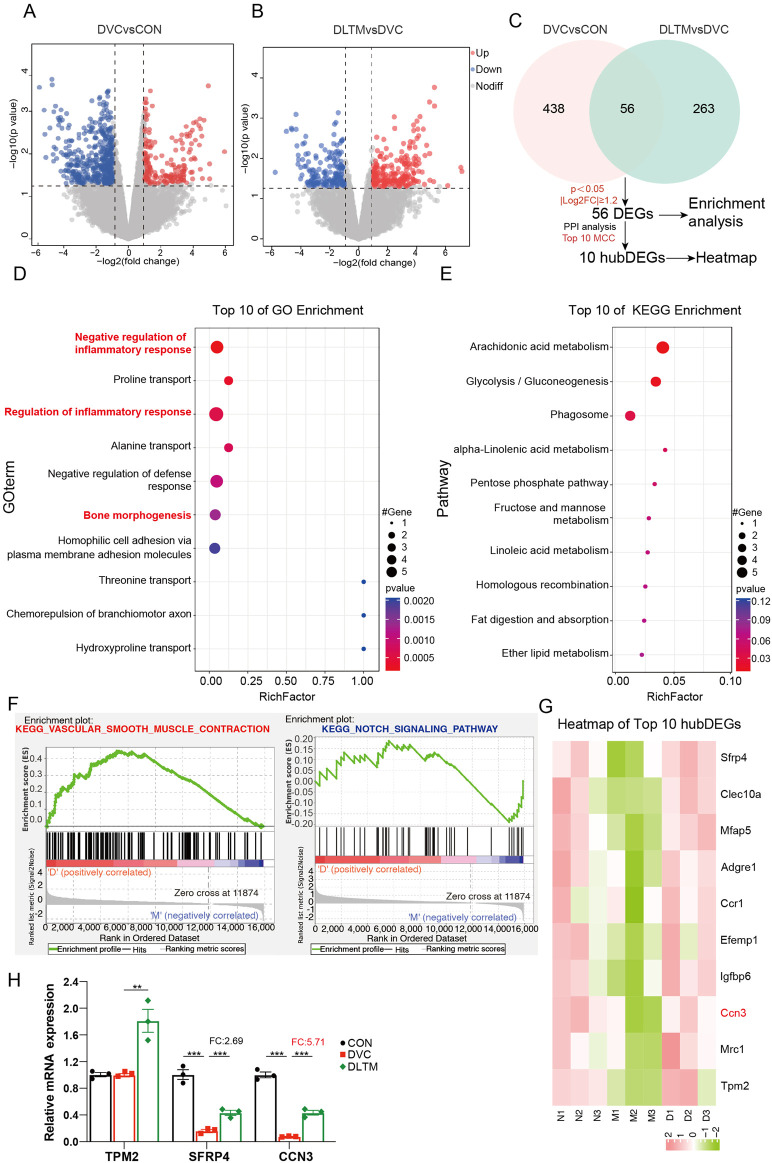
Transcriptome sequencing reveals the mechanism of DLTM against diabetic VC. **(A)** Volcano plot of transcriptome sequencing showing DEGs between the DVC group and CON group, with thresholds of *P* < 0.05 and |log2 (Fold Change) | ≥ 1.2; **(B)** Volcano plot of transcriptome sequencing showing DEGs between the DLTM group and DVC group, with thresholds of *P* < 0.05 and |log2 (Fold Change) | ≥ 1.2; **(C)** Venn diagram showing DEGs between DVC and CON groups, DLTM and DVC groups, with enrichment analysis and PPI analysis of overlapping DEGs to identify core DEGs; **(D)** GO enrichment analysis results of overlapping DEGs; **(E)** KEGG enrichment analysis results of overlapping DEGs; **(F)** GSEA analysis results of transcripts; **(G)**. Heatmap of Top10 core DEGs, **(H)** The mRNA expression levels of calcium-related hubDEGs among the CON, DVC, and DLTM groups (n=3). N: CON group, M: DVC group, D: DLTM group. ^*^ *P* < 0.05, ^**^ *P* < 0.01, ^***^ *P* < 0.001.

To explore the specific molecular mechanisms of DLTM against VC, transcriptome sequencing analysis was conducted to identify differentially expressed transcripts. Considering that high-dose DLTM exhibited an optimal therapeutic effect comparable to the positive drug Dapagliflozin, transcriptome sequencing was performed on aorta samples from the DLTM-H group. Post DLTM treatment, significant changes occurred in the transcriptome profile of rat aorta, revealing a total of 813 DEGs. Among them, 494 DEGs were identified between the DVC and CON groups, including 137 upregulated and 357 downregulated genes (Figure 4A). Alternatively, a total of 319 DEGs were found between the DLTM and DVC groups, featuring 175 upregulated and 144 downregulated genes (Figure 4B). Notably, 56 DEGs overlapped between the two groups (Figure 4C). Further enrichment analysis of the overlapping DEGs unveiled that DLTM-affected DEGs were predominantly enriched in negative regulation of inflammatory response, regulation of inflammatory response, and bone morphogenesis (Figure 4D). Kyoto Encyclopedia of Genes and Genomes (KEGG) enrichment analysis highlighted close associations with pathways such as glycolipid metabolism (Figure 4E). To prevent the enrichment results from being biased by considerable upregulation or downregulation of genes and potentially missing out on DEGs of significant biological importance, gene set enrichment analysis (GSEA) was performed on the entire transcriptome (Figure 4F). The results revealed a significant upregulation of genes related to vascular smooth muscle contraction in the DLTM group, consistent with phenotypic observations. This finding suggests that DLTM may promote vascular smooth muscle contraction by upregulating VSMC contractile proteins and restoring the contractile phenotype. Notably, significant enrichment of the NOTCH signaling pathway, a calcium-related pathway, was observed among different groups, which was notably downregulated following DLTM treatment. NOTCH signaling is a highly conserved pathway crucial for immune cell differentiation and homeostasis (Gallenstein et al., 2023). Previous research indicates that activation of the NOTCH signaling pathway can stimulate pro-inflammatory macrophages, thereby initiating subsequent inflammatory responses and facilitating the osteogenic differentiation and calcification of VSMCs (Kang et al., 2024). Furthermore, among the calcium-related hub differentially expressed genes (hubDEGs), CCN3 garnered attention (Figure 4G), with PCR results confirming it as the most significantly affected calcium-related hubDEG by DLTM (Figure 4H). CCN3/NOV (nephroblastoma overexpressed), part of the cellular communication network (CCN) protein family, serves as an essential regulator in bone remodeling, with high expression observed in endothelial cells, smooth muscle cells, fibroblasts, and chondrocytes (Ouellet and Siegel, 2012; Peng et al., 2021). Previous research has demonstrated that CCN3 can play an important regulatory role across various cell types by modulating the NOTCH signaling pathway, including inhibiting cellular osteogenic differentiation (Minamizato et al., 2007; Ouellet and Siegel, 2012; Luan et al., 2023). Combining these findings with the results of Gene Ontology (GO) enrichment analysis, it is speculated that DLTM may suppress the progression of DVC by regulating the CCN3/NOTCH signaling axis to modulate inflammation.

### 3.5 Molecular docking indicates superior binding affinity between the metabolites of DLTM and CCN3/NOTCH signaling pathway

**FIGURE 5:**
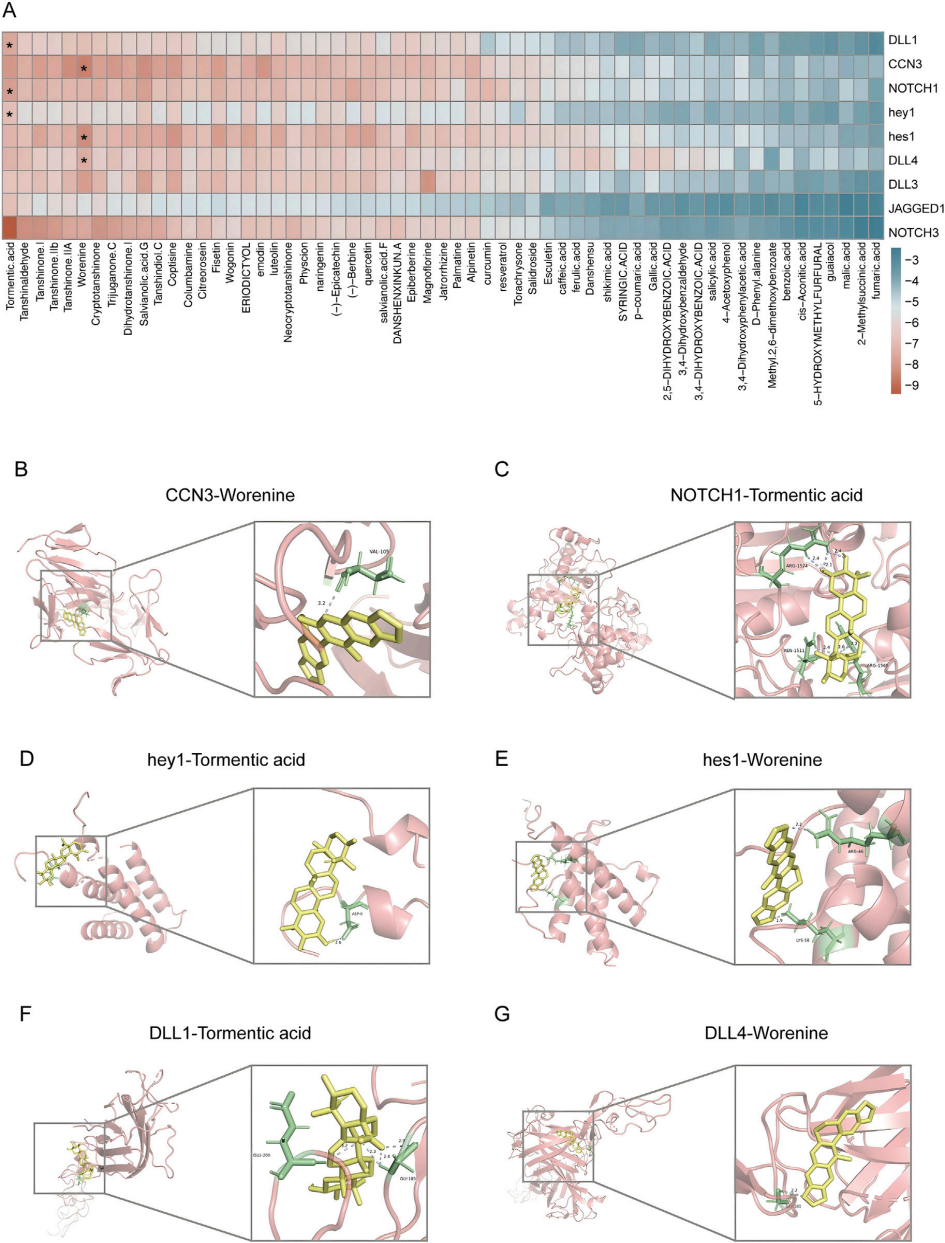
Metabolites of DLTM exhibited good binding affinities to molecules in CCN3/NOTCH signaling pathway. **(A)** Heatmap of binding energies between potentially effective metabolites of DLTM and molecules in CCN3 and NOTCH signaling pathways (see Supplementary Table S6 for details); **(B–G)** Molecular docking images showing the DLTM metabolites with the highest binding energies to CCN3 and various NOTCH signaling molecules, **(B)** CCN3- Worenine (−8.9 kcal/mol); **(C)** NOTCH1- Tormentic acid (−8.2 kcal/mol); **(D)** hey1- Tormentic acid (−7.2 kcal/mol); **(E)** hes1 (−8.6 kcal/mol)- Worenine; **(F)** DLL1-Tormentic acid-(−7.9 kcal/mol); **(G)** DLL4-Worenine (−7.7 kcal/mol).

The above results suggested that the mechanism by which DLTM treated DVC was related to the regulation of the CCN3/NOTCH signaling pathway. However, the interactions between the metabolites of DLTM and the molecules of the CCN3/NOTCH signaling pathway remained unclear. Therefore, molecular docking was performed using AutoDock to investigate the interactions between the potentially effective metabolites of DLTM and CCN3 as well as the classic NOTCH signaling molecules. The metabolites of DLTM were screened using Lipinski’s rule of five and SwissADME, resulting in potentially effective 59 metabolites for molecular docking (Supplementary Tables S4, S5). It is generally believed that when the binding energy is less than zero, the metabolite can spontaneously bind to the protein, with lower binding energy indicating a higher likelihood of interaction. The results showed that the average binding energy of the metabolites of DLTM with CCN3 and NOTCH signaling pathway proteins was −6.06 kcal/mol, with 69.30% of the molecules having binding energies < −5.25 kcal/mol and 26.93% having binding energies < −7 kcal/mol. The average binding energies for CCN3, NOTCH1, DLL4, DLL3, NOTCH3, DLL1, hes1, and hey1 were consistently below −5.25 kcal/mol (Figure 5A), suggesting superior binding activity with most DLTM metabolites (detailed in Supplementary Table S6). The 3D structure results further indicated that several main metabolites of DLTM, including worenine and tormentic acid, could well occupy the binding sites of CCN3 and classic NOTCH signaling molecules such as DLL1, DLL4, NOTCH1, hes1, and hey1 (Figures 5B–G). Conversely, the NOTCH ligand JAGGED1 exhibited lower binding energies with most DLTM metabolites, and NOTCH3 showed lower average binding energy than NOTCH1. Hence, it is hypothesized that CCN3, DLL1, DLL4, NOTCH1, and downstream transcription factors hes1 and hey1 may serve as key targets for DLTM in combating DVC.

To further evaluate the stability of the molecular docking results, we selected Worenine, the metabolite with the lowest average binding energy, and performed molecular dynamics simulations with the top two target proteins CCN3 and hes1, ranked by binding energy. The RMSD curve of the CCN3-Worenine complex remained relatively stable throughout the 0–100 ns simulation, fluctuating around 3.3 Å, and reached equilibrium after 80 ns (Supplementary Figure S2A). During the simulation, the Rg and SASA of the CCN3-Worenine complex remained relatively stable, indicating that the complex maintained a structurally stable and compact form (Supplementary Figures S2B, C). In contrast, the Rg of the hes1-Worenine complex exhibited noticeable fluctuations during the first 60 ns, and its SASA showed slight variations between 40 and 60 ns, suggesting that the binding of Worenine induced minor conformational changes in the protein, though these changes were within an acceptable range (Supplementary Figures S2B, C). Additionally, both the CCN3-Worenine and hes1-Worenine complexes maintained at least one hydrogen bond throughout the simulation, indicating stable binding between Worenine and the two target proteins (Supplementary Figure S2D). RMSF analysis suggested that the CCN3-Worenine complex exhibited higher stability, whereas the Hes1-Worenine complex was more flexible (Supplementary Figures S2E, F). Subsequently, we conducted MM/PBSA calculations to ascertain the binding energies of the two complexes and to pinpoint the key amino acids that substantially contribute to the binding affinity (Supplementary Figure S2G). These findings confirm the stable interaction of the DLTM metabolite Worenine with the target proteins CCN3 and hes1, further validating the molecular docking results. Combining the results of transcriptome, we speculated that the CCN3/NOTCH signaling pathway is a key pathway through which DLTM exerts its anti-DVC effects.

### 3.6 DLTM inhibits the release of inflammatory factors by regulating the CCN3/NOTCH signaling axis

**FIGURE 6:**
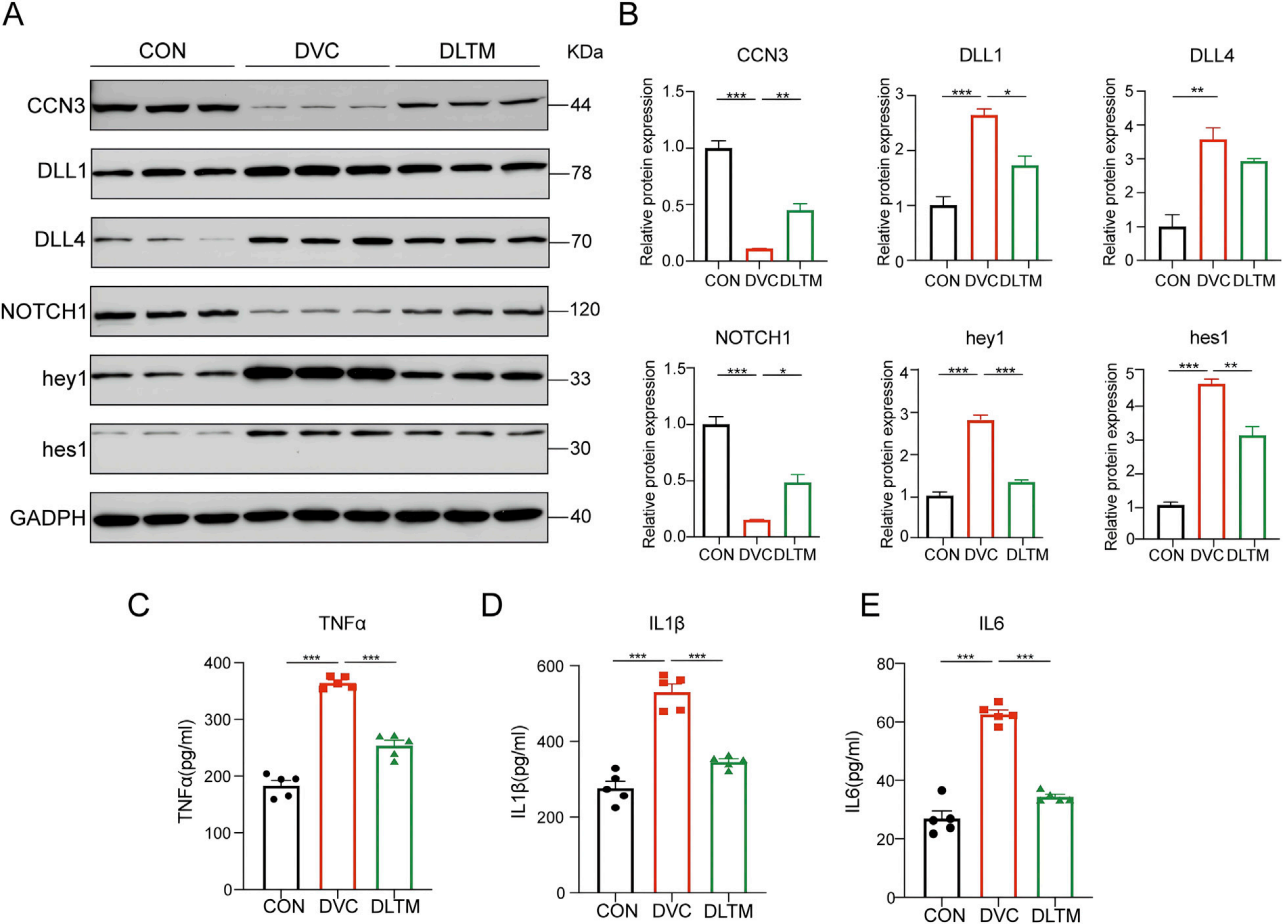
DLTM inhibits the release of inflammatory factors by regulating CCN3/NOTCH signal axis. **(A)** Representative Western blot images of CCN3, DLL1, DLL4, NOTCH1, hey1, hes1 expression in the aortas of rats from the CON, DVC, and DLTM groups; **(B)** Quantitative analysis of CCN3, DLL1, DLL4, NOTCH1, hey1, hes1 expression (n = 3); **(C)** Serum TNFα levels in rats from the CON, DVC, and DLTM groups (n = 5); **(D)** Serum IL1β levels in rats from the CON, DVC, and DLTM groups (n = 5); **(E)** Serum IL6 levels in rats from the CON, DVC, and DLTM groups (n = 5). ^*^ *P* < 0.05, ^**^ *P* < 0.01, ^***^ *P* < 0.001.

To verify the effect of DLTM on the CCN3/NOTCH signaling axis *in vivo*, the expression levels of CCN3 and the NOTCH signaling proteins with high binding activity in the aortas of rats from different groups were examined. The results showed that, compared to the CON group, the DVC group exhibited substantially decreased expressions of CCN3 and NOTCH1, while the expressions of NOTCH signaling ligands DLL1, DLL4, and downstream transcription factors hey1 and hes1 were significantly elevated. DLTM intervention effectively upregulated the expressions of CCN3 and NOTCH1 and inhibited the activation of DLL1 and downstream transcription factors hey1 and hes1. Nevertheless, it exerted no significant effect on DLL4 (Figures 6A, B, *P* < 0.001, *P* < 0.01, *P* < 0.05). Furthermore, ELISA was used to detect the levels of inflammatory factors in serum. The results showed that, compared to the CON group, the DVC group had significantly increased serum levels of TNFα, IL1β, and IL6. Meanwhile, DLTM intervention reversed these trends, reducing the release of the inflammatory factors TNFα, IL1β, and IL6 (Figures 6C–E, *P* < 0.001). These results collectively indicate that DLTM can exert anti-inflammatory effects by activating CCN3, inhibiting the expressions of NOTCH signaling ligand DLL1 and downstream transcription factors hey1 and hes1, and suppressing the activation of the NOTCH signaling pathway.

## 4 Discussion

VC is a prevalent and life-threatening complication in diabetic patients, adversely affecting clinical outcomes. As an intermediary condition between diabetes and the subsequent damage to target organs, the clinical prevention and treatment of VC remains largely unaddressed. TCM has advantages in preventive treatment of disease, which is beneficial for improving VC, an early vascular injury. DLTM, a TCM prescription, has been successfully used to treat diabetic vascular complications in clinical practice. In this study, we first identified the main metabolites of DLTM and confirmed that it can inhibit inflammatory responses by regulating the CCN3/NOTCH signaling axis, preventing VSMCs from transitioning from a contractile phenotype to an osteogenic phenotype, thereby alleviating DVC.

Diabetes-induced imbalances in glucose and calcium-phosphorus metabolism are key risk factors for pathological VC (Yahagi et al., 2017; Zhang et al., 2023). Studies show that Patients with VC exhibit varying degrees of elevated serum calcium and phosphorus levels, with serum calcium levels significantly positively correlated with coronary and abdominal aortic calcification (Wang et al., 2010; Zhu et al., 2024). In this study,we found that DVC rats exhibited significant disturbances in glucose and calcium-phosphorus metabolism, with FBG and serum calcium levels significantly elevated, corresponding to their severe vascular damage. However, since we established the DVC model by inducing calcium overload, no significant changes in serum phosphorus were observed in the short term. Interventions with different doses of DLTM effectively improved serum calcium levels but had no significant effect on blood glucose. This suggests that the therapeutic effect of DLTM on DVC is independent of glycemic control and is related to calcium balance regulation.

The osteogenic transformation of VSMC is a core pathological process in DVC, and is closely associated with the activation of various metabolites in the immune-inflammatory response process (Yahagi et al., 2017). Inflammatory response is one of the key mechanisms linking diabetes and VC. This is because metabolic disorders induced by diabetes, such as hyperglycemia and insulin resistance, promote the release of various inflammatory mediators, thereby establishing an inflammatory microenvironment conducive to the osteogenic transformation of VSMC (Chen et al., 2020; Gora et al., 2021; Xue et al., 2023). Additionally, clinical and animal experiments have shown that focal arterial inflammation in the vascular system precedes atherosclerosis or calcification formation at the same site (Aikawa et al., 2007; Abdelbaky et al., 2013; Joshi et al., 2016). Inflammatory factors and related transcription factors can directly promote VC or indirectly facilitate calcification by reducing the levels of calcification inhibitors. For instance, TNFα can activate the NF-κB pathway to upregulate osteogenic genes Msx2 and ALP, promoting the osteogenic transdifferentiation and calcification of VSMCs (Lee et al., 2010). Toll-like receptor 2 (TLR2)-mediated NF-κB activation induces VC in atherosclerosis by activating p38 and ERK1/2 signaling and inhibiting osteoprotegerin (OPG) expression (Lee et al., 2019). Moreover, the irreversible formation and accumulation of advanced glycation end products (AGEs) are crucial mechanisms exacerbating systemic inflammation and promoting diabetic VC (Kay et al., 2016). Research indicates that AGE/RAGE can activate inflammatory-related signaling pathways such as p38-MAPK, TGF-β, and NF-κB, upregulate osteogenic proteins Msx2 and BMP2, inhibit the contractile phenotype of VSMCs, and induce osteogenic transformation and VC in VSMCs (Tanikawa et al., 2009; Sakaguchi et al., 2011; Ndip et al., 2014). These studies collectively demonstrate that inflammation is key in triggering and driving DVC, playing a continuous role throughout its development. Regulating inflammation-related pathways to protect against DVC holds significant importance for new drug development and the repurposing of existing drugs.

In recent years, numerous studies have confirmed that some antidiabetic drugs can alleviate VC by inhibiting inflammatory responses (Ghosh et al., 2020). For instance, SGLT2 inhibitors such as canagliflozin and empagliflozin, which are beneficial for both heart and kidney health, have been shown to inhibit the osteogenic transformation of VSMCs and VC by suppressing NLRP3 inflammasome activation (Chen et al., 2023; Li X. et al., 2024). Traditional antidiabetic drugs such as metformin have been found to reduce pro-inflammatory factors TNF-α and IL-1β levels in clinical and experimental models, lowering clinical arterial calcification scores and inhibiting VC development (Mary et al., 2017; Araújo et al., 2017; Lu et al., 2019). Additionally, metabolites in certain TCMs, such as tanshinone IIA and 6-gingerol, have been discovered to possess anti-inflammatory and anti-VC effects (Chen et al., 2020; Zhong et al., 2022). In this study, we found that DLTM effectively inhibited the osteogenic differentiation of VSMC and DVC *in vivo*. High-throughput transcriptomics and DEGs analysis suggest that its mechanism may involve promoting vascular smooth muscle contraction and inhibiting inflammatory responses, with the NOTCH signaling pathway likely being a key pathway.

The NOTCH signaling pathway is a critical intercellular communication route that controls cell fate. In mammals, four NOTCH receptors (NOTCH1-4) and five ligands (JAGGED1-2, DLL1, 3, 4) have been identified. These receptors and ligands mediate classical signal transduction, including those related to calcification and inflammation, by triggering a series of cleavage events and nuclear CSL-dependent transcription. NOTCH signaling is essential for BMP-induced osteogenic transformation of VSMCs. The intracellular domain of NOTCH1, N1-ICD, drives the expression of the osteogenic gene Msx2 by interacting with the downstream transcription factor Smad1 of BMP2, thereby inducing VSMC osteogenic transformation and calcification (Shimizu et al., 2011). Moreover, the activation of NOTCH signaling can exacerbate atherosclerosis and VC formation in mice by promoting macrophage activation and inflammatory factor release (Nakano et al., 2019). Similarly, our study found that NOTCH signaling was significantly upregulated in DVC. Combined with enrichment analysis results, it is speculated that NOTCH signaling may promote VC development by exacerbating inflammatory responses. CCN3 is one of the non-classical ligands of NOTCH1 and is involved in the regulation of osteoblast differentiation (Suresh et al., 2013). Previous studies have shown that CCN3 can regulate NOTCH1 signaling in myoblasts (Zhang and Wang, 2011), osteoblasts (Minamizato et al., 2007), and VSMCs (Abe and Yan, 2010) to participate in cellular osteogenic transformation. In our study, CCN3 was not only identified as a hub DEG in the anti-DVC effect of DLTM but was also the most influenced gene by DLTM, exhibiting a negative correlation with NOTCH signaling expression. Consequently, it is speculated that DLTM may exert its anti-DVC effects by modulating the CCN3/NOTCH signaling axis to inhibit inflammatory responses.

**FIGURE 7:**
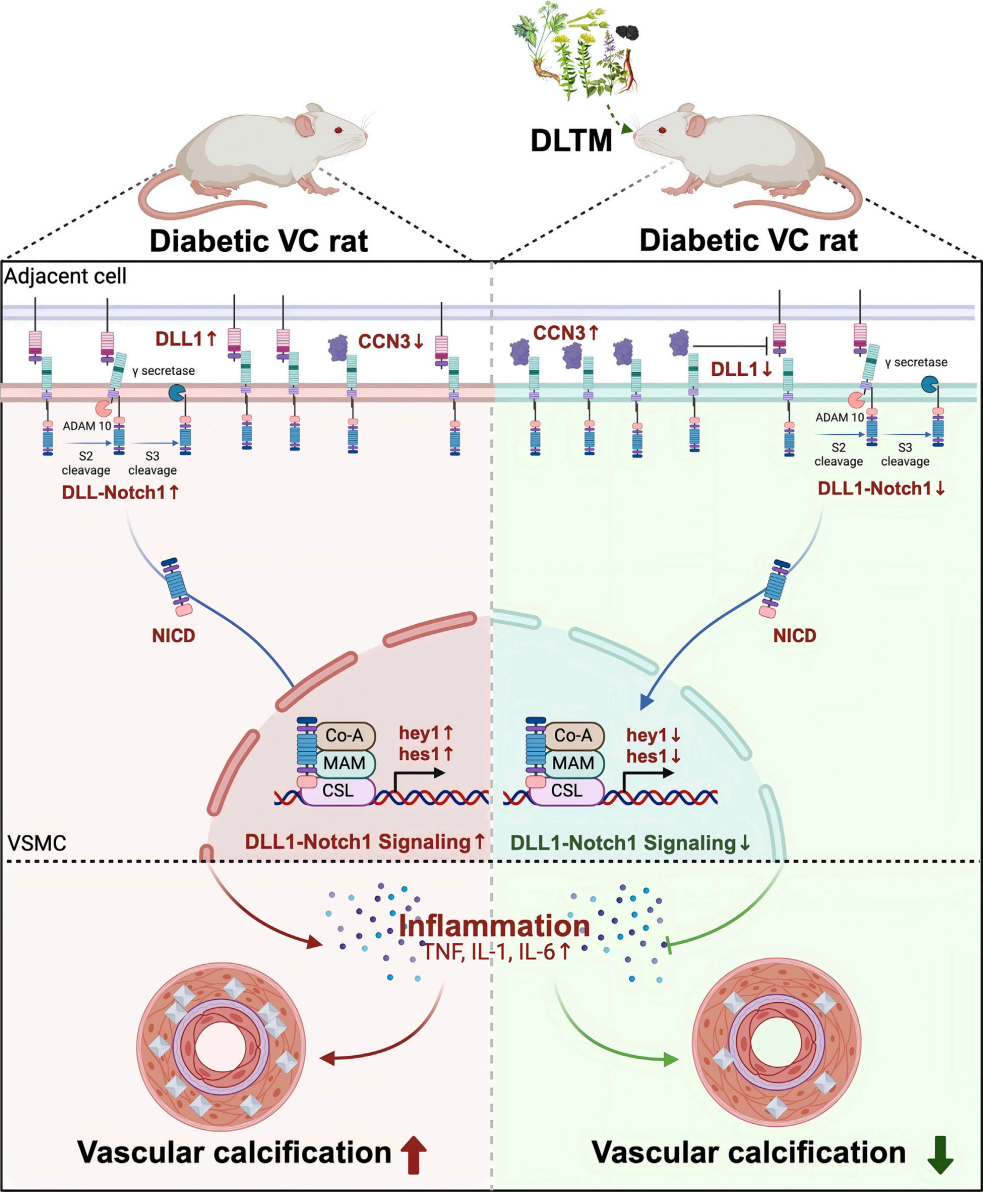
The mechanism diagram of DLTM against diabetic VC.

Molecular docking and molecular dynamics simulations suggested that the metabolites of DLTM could effectively bind to CCN3 and NOTCH signaling molecules, exhibiting stable interactions and structural compatibility. *In vivo* experiments further confirmed that DLTM significantly upregulated CCN3 expression. Nevertheless, within the NOTCH signaling pathway, DLTM activated NOTCH1 expression while simultaneously inhibiting the expressions of the ligand DLL1 and downstream transcription factors hes1 and hey1. NOTCH signal transduction requires the binding of ligands to NOTCH receptors to initiate a series of cleavage events and releasing the soluble intracellular domain (NICD). The NICD then enters the nucleus and interacts with CSL to form a multiprotein-DNA complex that promotes the transcription of NOTCH target genes (Shi et al., 2024). Although CCN3 can interact with NOTCH1 and participate in downstream NOTCH signaling, this CCN3-NOTCH1 association does not depend on Ca^2+^ and does not affect the interaction between other ligands and NOTCH1. In addition, CCN3 primarily binds to the extracellular domain of NOTCH1 (NECD) without triggering NOTCH1 cleavage or NICD release (Sakamoto et al., 2002), which may result in a weaker effect compared to the influence of the DLL1-NOTCH interaction on downstream NOTCH target genes. Thus, it is speculated that although DLTM activates CCN3 and upregulates NOTCH1 expression, the activation of CCN3 also inhibits the expression of the NOTCH ligand DLL1, reducing the binding of DLL1 to NOTCH1 and thereby inhibiting the activation of downstream transcription factors hey1 and hes1. Despite the upregulation of NOTCH1, the overall NOTCH signaling is inhibited. Previous research has confirmed that CCN3 can downregulate the NOTCH signaling transcription factor hey1 and inhibit both early and late osteogenic differentiation of embryonic fibroblasts by competitively inhibiting the expression of the NOTCH1 ligand DLL1 (Su et al., 2018). Our findings revealed that DLTM upregulated CCN3 while inhibiting the activation of the NOTCH ligand DLL1 and downstream transcription factors hey1 and hes1. The downregulation of NOTCH signaling further reduced the release of downstream inflammatory factors TNFα, IL1β, and IL6, thereby effectively alleviating inflammation and DVC (Figure 7).

Currently, several TCM formulas have been proven effective in treating DVC. For example, Shenqi Compound has been demonstrated to inhibit glucolipid toxicity, alleviate apoptosis and phenotypic transformation of VSMC, and improve DVC by regulating inflammatory responses, extracellular matrix remodeling, and the Hippo-YAP signaling pathway (Yang et al., 2023). Chinese patent drug Danzhi Jiangtang capsule has also been found to inhibit the osteogenic transformation of VSMCs *in vitro* by suppressing the synthesis of β-catenin protein in the Wnt signaling pathway (Ni et al., 2023). Our study comprehensively evaluated the therapeutic effects of DLTM on DVC through gross staining, calcification markers, and the expression of osteogenic and contractile proteins, providing new evidence to support the use of TCM in the prevention and treatment of DVC. Notably, compared to existing TCM treatments, DLTM exhibits a more unique therapeutic mechanism, particularly in its regulatory effects on the CCN3/NOTCH pathway. The role of CCN proteins in vascular diseases is receiving increasing attention (Attramadal et al., 2024), which highlights the significant potential of DLTM in regulating vascular health. However, due to the presence of multiple metabolites in DLTM, this study only used molecular docking to predict their potential binding of with the CCN3/NOTCH pathway, and the lack of metabolite-protein binding evaluation and gene knockout studies to verify the specific roles of CCN3 and NOTCH signaling-related molecules in DVC within this study, further research is needed to explore the active metabolites and detailed mechanisms of DLTM.

Additionally, while this study used a DVC model induced by STZ and VitD3 to provide important insights into the treatment of DVC by DLTM, future research should consider incorporating diet-induced type 2 diabetes models to comprehensively evaluate the efficacy of DLTM across DVC. Conducting studies with longer intervention periods will also help fully assess the effectiveness and safety of DLTM, providing more evidence for its clinical development and application.

## 5 Conclusion

The results of this study suggest that DLTM can effectively inhibit DVC through the regulation of the CCN3/NOTCH signaling axis to suppress inflammatory responses.

## Data Availability

The original contributions presented in the study are publicly available. This data can be found here: http://www.ncbi.nlm.nih.gov/bioproject/1200899.

## Glossary

AGEs: Advanced glycation end products
ALP: Alkaline phosphatase
BV: Bed volumes
CCN: Cellular communication network
CSL: CBF1/suppressor of hairless/Lag1
DEGs: Differentially expressed genes
DLTM: Danlian-Tongmai formula
DVC: Diabetic vascular calcification
ELISA: Enzyme-linked immunosorbent assay
ESI: Electrospray ionization
GO: Gene Ontology
GI: Gastrointestinal
GSEA: Gene set enrichment analysis
hubDEGs: Hub differentially expressed genes
IL: Interleukin
MTB: Methylthymol blue
NIH: National Institutes of Health
NICD: Notch intracellular domain
NECD: Notch extracellular domain
PVDF: Polyvinylidene fluoride
OPG: Osteoprotegerin
qPCR: Quantitative real-time polymerase chain reaction
SD: Sprague Dawley
STZ: Streptozotocin
SDS-PAGE: Sodium dodecyl sulfate-polyacrylamide gel electrophoresis
T2DM: Type 2 diabetes mellitus
TLR2: Toll-like receptor 2
UHPLC-MS: Ultra-high-performance liquid chromatography-mass spectrometry
VC: Vascular calcification
VitD3: Vitamin D3
VSMCs: Vascular smooth muscle cells

## References

[B1] Abdelbaky A. Corsini E. Figueroa A. L. Fontanez S. Subramanian S. Ferencik M. (2013). Focal arterial inflammation precedes subsequent calcification in the same location: a longitudinal FDG-PET/CT study. Circ. Cardiovasc. imaging 6, 747–754. 10.1161/CIRCIMAGING.113.000382 23833282

[B2] Abe J. Yan C. (2010). CCNs-Notch signaling in vascular smooth muscle cells; Good or Bad? Arterioscler. Thromb. Vasc. Biol. 30, 667–668. 10.1161/ATVBAHA.109.202713 20237327 PMC3008587

[B3] Abraham M. J. Murtola T. Schulz R. Páll S. Smith J. C. Hess B. (2015). GROMACS: high performance molecular simulations through multi-level parallelism from laptops to supercomputers. SoftwareX 1–2, 19–25. 10.1016/j.softx.2015.06.001

[B4] Aikawa E. Nahrendorf M. Figueiredo J.-L. Swirski F. K. Shtatland T. Kohler R. H. (2007). Osteogenesis associates with inflammation in early-stage atherosclerosis evaluated by molecular imaging *in vivo* . Circulation 116, 2841–2850. 10.1161/CIRCULATIONAHA.107.732867 18040026

[B5] Araújo A. A. de Pereira A. de S. B. F. Medeiros C. A. C. X. de Brito G. A. de C. Leitão R. F. de C. Araújo L. de S. (2017). Effects of metformin on inflammation, oxidative stress, and bone loss in a rat model of periodontitis. PLoS One 12, e0183506. 10.1371/journal.pone.0183506 28847008 PMC5573680

[B6] Attramadal H. Weiskirchen R. Perbal B. (2024). Report on the 12th international workshop on the CCN family of genes, Oslo, June 20-23, 2024. J. Cell Commun. Signal 18, e12049. 10.1002/ccs3.12049 39524142 PMC11544635

[B7] Bassa L. M. Jacobs C. Gregory K. Henchey E. Ser-Dolansky J. Schneider S. S. (2016). Rhodiola crenulata induces an early estrogenic response and reduces proliferation and tumorsphere formation over time in MCF7 breast cancer cells. Phytomedicine 23, 87–94. 10.1016/j.phymed.2015.11.014 26850689

[B8] Bennett R. A. Pegg A. E. (1981). Alkylation of DNA in rat tissues following administration of streptozotocin. Cancer Res. 41, 2786–2790. 6454479

[B9] Chen A. Lan Z. Li L. Xie L. Liu X. Yang X. (2023). Sodium-glucose cotransporter 2 inhibitor canagliflozin alleviates vascular calcification through suppression of nucleotide-binding domain, leucine-rich-containing family, pyrin domain-containing-3 inflammasome. Cardiovasc Res. 119, 2368–2381. 10.1093/cvr/cvad119 37523743

[B10] Chen T.-C. Yen C.-K. Lu Y.-C. Shi C.-S. Hsieh R.-Z. Chang S.-F. (2020). The antagonism of 6-shogaol in high-glucose-activated NLRP3 inflammasome and consequent calcification of human artery smooth muscle cells. Cell Biosci. 10, 5. 10.1186/s13578-019-0372-1 31938471 PMC6953308

[B11] Déciga-Campos M. González-Trujano M. E. Ventura-Martínez R. Montiel-Ruiz R. M. Ángeles-López G. E. Brindis F. (2016). Antihyperalgesic activity of Rhodiola rosea in a diabetic rat model. Drug Dev. Res. 77, 29–36. 10.1002/ddr.21289 26763184

[B12] Ferket B. S. Hunink M. G. M. Masharani U. Max W. Yeboah J. Burke G. L. (2022). Lifetime cardiovascular disease risk by coronary artery calcium score in individuals with and without diabetes: an analysis from the multi-ethnic study of atherosclerosis. Diabetes Care 45, 975–982. 10.2337/dc21-1607 35168253 PMC9114718

[B13] Furman B. L. (2021). Streptozotocin-induced diabetic models in mice and rats. Curr. Protoc. 1, e78. 10.1002/cpz1.78 33905609

[B14] Gallenstein N. Tichy L. Weigand M. A. Schenz J. (2023). Notch signaling in acute inflammation and sepsis. Int. J. Mol. Sci. 24, 3458. 10.3390/ijms24043458 36834869 PMC9967996

[B15] Ghosh S. Luo D. He W. Chen J. Su X. Huang H. (2020). Diabetes and calcification: the potential role of anti-diabetic drugs on vascular calcification regression. Pharmacol. Res. 158, 104861. 10.1016/j.phrs.2020.104861 32407954

[B16] Gora I. M. Ciechanowska A. Ladyzynski P. (2021). NLRP3 inflammasome at the interface of inflammation, endothelial dysfunction, and type 2 diabetes. Cells 10, 314. 10.3390/cells10020314 33546399 PMC7913585

[B17] Harrach M. F. Drossel B. (2014). Structure and dynamics of TIP3P, TIP4P, and TIP5P water near smooth and atomistic walls of different hydroaffinity. J. Chem. Phys. 140, 174501. 10.1063/1.4872239 24811640

[B18] Heath J. M. Sun Y. Yuan K. Bradley W. E. Litovsky S. Dell’Italia L. J. (2014). Activation of AKT by O-linked N-acetylglucosamine induces vascular calcification in diabetes mellitus. Circ. Res. 114, 1094–1102. 10.1161/CIRCRESAHA.114.302968 24526702 PMC4030422

[B19] Huang X. Wang Y. Qiu Y. Shi Q. Sun D. Yang J. (2022). Resveratrol ameliorates high-phosphate-induced VSMCs to osteoblast-like cells transdifferentiation and arterial medial calcification in CKD through regulating Wnt/β-catenin signaling. Eur. J. Pharmacol. 925, 174953. 10.1016/j.ejphar.2022.174953 35483665

[B20] Jiang H. Li L. Zhang L. Zang G. Sun Z. Wang Z. (2022). Role of endothelial cells in vascular calcification. Front. Cardiovasc Med. 9, 895005. 10.3389/fcvm.2022.895005 35928939 PMC9343736

[B21] Jin Z.-H. Gao P. Liu Z.-T. Jin B. Song G.-Y. Xiang T.-Y. (2020). Composition of ophiopogon polysaccharide, notoginseng total saponins and rhizoma coptidis alkaloids inhibits the myocardial apoptosis on diabetic atherosclerosis rabbit. Chin. J. Integr. Med. 26, 353–360. 10.1007/s11655-018-3014-2 30328567

[B22] Jo S. Kim T. Iyer V. G. Im W. (2008). CHARMM-GUI: a web-based graphical user interface for CHARMM. J. Comput. Chem. 29, 1859–1865. 10.1002/jcc.20945 18351591

[B23] Joshi F. R. Rajani N. K. Abt M. Woodward M. Bucerius J. Mani V. (2016). Does vascular calcification accelerate inflammation? a substudy of the dal-PLAQUE trial. J. Am. Coll. Cardiol. 67, 69–78. 10.1016/j.jacc.2015.10.050 26764069

[B24] Kang J.-H. Kawano T. Murata M. Toita R. (2024). Vascular calcification and cellular signaling pathways as potential therapeutic targets. Life Sci. 336, 122309. 10.1016/j.lfs.2023.122309 38042282

[B25] Kay A. M. Simpson C. L. Stewart J. A. (2016). The role of AGE/RAGE signaling in diabetes-mediated vascular calcification. J. Diabetes Res. 2016, 6809703. 10.1155/2016/6809703 27547766 PMC4980539

[B26] Kono R. Okuno Y. Inada K. Tokuda A. Hashizume H. Yoshida M. (2011). A Prunus mume extract stimulated the proliferation and differentiation of osteoblastic MC3T3-E1 cells. Biosci. Biotechnol. Biochem. 75, 1907–1911. 10.1271/bbb.110264 21979066

[B27] Lee G.-L. Yeh C.-C. Wu J.-Y. Lin H.-C. Wang Y.-F. Kuo Y.-Y. (2019). TLR2 promotes vascular smooth muscle cell chondrogenic differentiation and consequent calcification via the concerted actions of osteoprotegerin suppression and IL-6-mediated RANKL induction. Arterioscler. Thromb. Vasc. Biol. 39, 432–445. 10.1161/ATVBAHA.118.311874 30626205

[B28] Lee H.-L. Woo K. M. Ryoo H.-M. Baek J.-H. (2010). Tumor necrosis factor-alpha increases alkaline phosphatase expression in vascular smooth muscle cells via MSX2 induction. Biochem. Biophysical Res. Commun. 391, 1087–1092. 10.1016/j.bbrc.2009.12.027 20004646

[B29] Lee S. J. Lee I.-K. Jeon J.-H. (2020). Vascular calcification—new insights into its mechanism. Int. J. Mol. Sci. 21, 2685. 10.3390/ijms21082685 32294899 PMC7216228

[B30] Lei M.-H. Wu Y.-L. Chung S.-L. Chen C.-C. Chen W.-C. Hsu Y.-C. (2021). Coronary artery calcium score predicts long-term cardiovascular outcomes in asymptomatic patients with type 2 diabetes. J. Atheroscler. Thromb. 28, 1052–1062. 10.5551/jat.59386 33162430 PMC8560843

[B31] Li J.-B. Xu L.-J. Dong H. Huang Z.-Y. Zhao Y. Chen G. (2013). Effects of Chinese Fructus Mume formula and its separated prescription extract on insulin resistance in type 2 diabetic rats. J. Huazhong Univ. Sci. Technol. Med. Sci. 33, 877–885. 10.1007/s11596-013-1215-7 24337852

[B32] Li L. Liu H. Chai Q. Wei J. Qin Y. Yang J. (2024a). Dapagliflozin targets SGLT2/SIRT1 signaling to attenuate the osteogenic transdifferentiation of vascular smooth muscle cells. Cell Mol. Life Sci. 81, 448. 10.1007/s00018-024-05486-8 39520538 PMC11550308

[B33] Li L. Zheng G. Cao C. Cao W. Yan H. Chen S. (2022). The ameliorative effect of berberine on vascular calcification by inhibiting endoplasmic reticulum stress. J. Cardiovasc Pharmacol. 80, 294–304. 10.1097/FJC.0000000000001303 35580317

[B34] Li X. Chen Z. Sun X. Yang Y. Jin H. Liu N. (2024b). Empagliflozin ameliorates vascular calcification in diabetic mice through inhibiting Bhlhe40-dependent NLRP3 inflammasome activation. Acta Pharmacol. Sin. 45, 751–764. 10.1038/s41401-023-01217-0 38172306 PMC10943241

[B35] Lipinski C. A. Lombardo F. Dominy B. W. Feeney P. J. (2001). Experimental and computational approaches to estimate solubility and permeability in drug discovery and development settings. Adv. Drug Deliv. Rev. 46, 3–26. 10.1016/s0169-409x(00)00129-0 11259830

[B36] Liu J. Sun X. Tao S. Liu H. Wu W. Liu W. (2024). Therapeutic effects and mechanisms of Modified Ma-Xing-Shi-Gan Decoction on Klebsiella pneumoniae-induced pneumonia in mice assessed by Multi-omics. J. Ethnopharmacol. 337, 118976. 10.1016/j.jep.2024.118976 39447714

[B37] Lu Y. Meng L. Ren R. Wang X. Sui W. Xue F. (2024). Paraspeckle protein NONO attenuates vascular calcification by inhibiting bone morphogenetic protein 2 transcription. Kidney Int. 105, 1221–1238. 10.1016/j.kint.2024.01.039 38417578

[B38] Lu Y. Wang Y. Weng T. Chen Z. Sun X. Wei J. (2019). Association between metformin use and coronary artery calcification in type 2 diabetic patients. J. Diabetes Res. 2019, 9484717. 10.1155/2019/9484717 31192264 PMC6525896

[B39] Luan Y. Zhang H. Ma K. Liu Y. Lu H. Chen X. (2023). CCN3/NOV regulates proliferation and neuronal differentiation in mouse hippocampal neural stem cells via the activation of the notch/PTEN/AKT pathway. Int. J. Mol. Sci. 24, 10324. 10.3390/ijms241210324 37373471 PMC10299577

[B40] Mao A. Li Z. Shi X. Zhang K. Kan H. Geng L. (2024). Complement factor C1q mediates vascular endothelial dysfunction in STZ-induced diabetic mice. Diabetes 73, 1527–1536. 10.2337/db23-0981 38869460

[B41] Mary A. Hartemann A. Liabeuf S. Aubert C. E. Kemel S. Salem J. E. (2017). Association between metformin use and below-the-knee arterial calcification score in type 2 diabetic patients. Cardiovasc. Diabetol. 16, 24. 10.1186/s12933-017-0509-7 28202017 PMC5311847

[B42] Mattison J. A. Wang M. Bernier M. Zhang J. Park S.-S. Maudsley S. (2014). Resveratrol prevents high fat/sucrose diet-induced central arterial wall inflammation and stiffening in nonhuman primates. Cell Metab. 20, 183–190. 10.1016/j.cmet.2014.04.018 24882067 PMC4254394

[B43] MEIm X.-D. Cao Y.-F. Che Y.-Y. Li J. Shang Z.-P. Zhao W.-J. (2019). Danshen: a phytochemical and pharmacological overview. Chin. J. Nat. Med. 17, 59–80. 10.1016/S1875-5364(19)30010-X 30704625

[B44] Minamizato T. Sakamoto K. Liu T. Kokubo H. Katsube K. Perbal B. (2007). CCN3/NOV inhibits BMP-2-induced osteoblast differentiation by interacting with BMP and Notch signaling pathways. Biochem. Biophys. Res. Commun. 354, 567–573. 10.1016/j.bbrc.2007.01.029 17250806

[B45] Nakano T. Katsuki S. Chen M. Decano J. L. Halu A. Lee L. H. (2019). Uremic toxin indoxyl sulfate promotes pro-inflammatory macrophage activation via the interplay of OATP2B1 and Dll4-Notch signaling. Circulation 139, 78–96. 10.1161/CIRCULATIONAHA.118.034588 30586693 PMC6311723

[B46] Ndip A. Wilkinson F. L. Jude E. B. Boulton A. J. M. Alexander M. Y. (2014). RANKL-OPG and RAGE modulation in vascular calcification and diabetes: novel targets for therapy. Diabetologia 57, 2251–2260. 10.1007/s00125-014-3348-z 25112376

[B75] Ni Y. Fang Z. Li J. (2023). Effect of Danzhi Jiangtang Capsule on regulating β-catenin protein on high phosphorus-induced calcification of vascular endothelial cell in lower limb in diabetic mice. J. Tianjin Univ. traditional Chin. Med. 42 (1), 81–86.

[B47] Okuno K. Torimoto K. Kuroda R. Cicalese S. M. Okuno Y. Kono R. (2023). Infused juice concentrate of Japanese plum Prunus mume attenuates inflammatory vascular remodeling in a mouse model of hypertension induced by angiotensin II. Hypertens. Res. 46, 1923–1933. 10.1038/s41440-023-01332-9 37308550

[B48] Ouellet V. Siegel P. M. (2012). CCN3 modulates bone turnover and is a novel regulator of skeletal metastasis. J. Cell Commun. Signal 6, 73–85. 10.1007/s12079-012-0161-7 22427255 PMC3368020

[B49] Peng L. Wei Y. Shao Y. Li Y. Liu N. Duan L. (2021). The emerging roles of CCN3 protein in immune-related diseases. Mediat. Inflamm. 2021, 5576059. 10.1155/2021/5576059 PMC8356028 34393649

[B50] Quaglino D. Boraldi F. Lofaro F. D. (2020). The biology of vascular calcification. Int. Rev. Cell Mol. Biol. 354, 261–353. 10.1016/bs.ircmb.2020.02.007 32475476

[B51] Reagan-Shaw S. Nihal M. Ahmad N. (2008). Dose translation from animal to human studies revisited. FASEB J. 22, 659–661. 10.1096/fj.07-9574LSF 17942826

[B52] Sakaguchi M. Murata H. Yamamoto K. Ono T. Sakaguchi Y. Motoyama A. (2011). TIRAP, an adaptor protein for TLR2/4, transduces a signal from RAGE phosphorylated upon ligand binding. PLoS One 6, e23132. 10.1371/journal.pone.0023132 21829704 PMC3148248

[B53] Sakamoto K. Yamaguchi S. Ando R. Miyawaki A. Kabasawa Y. Takagi M. (2002). The nephroblastoma overexpressed gene (NOV/ccn3) protein associates with Notch1 extracellular domain and inhibits myoblast differentiation via notch signaling pathway. J. Biol. Chem. 277, 29399–29405. 10.1074/jbc.M203727200 12050162

[B54] Shi Q. Xue C. Zeng Y. Yuan X. Chu Q. Jiang S. (2024). Notch signaling pathway in cancer: from mechanistic insights to targeted therapies. Signal Transduct. Target Ther. 9, 128. 10.1038/s41392-024-01828-x 38797752 PMC11128457

[B55] Shimizu T. Tanaka T. Iso T. Matsui H. Ooyama Y. Kawai-Kowase K. (2011). Notch signaling pathway enhances bone morphogenetic protein 2 (BMP2) responsiveness of Msx2 gene to induce osteogenic differentiation and mineralization of vascular smooth muscle cells. J. Biol. Chem. 286, 19138–19148. 10.1074/jbc.M110.175786 21471203 PMC3099727

[B56] Sohn E. Kim J. Kim C.-S. Jo K. Lee Y. M. Kim J. S. (2014). Root of Polygonum cuspidatum extract reduces progression of diabetes-induced mesangial cell dysfunction via inhibition of platelet-derived growth factor-BB (PDGF-BB) and interaction with its receptor in streptozotocin-induced diabetic rats. BMC Complement. Altern. Med. 14, 477. 10.1186/1472-6882-14-477 25495844 PMC4364577

[B57] Sohn E. Kim J. Kim C.-S. Lee Y. M. Kim J. S. (2016). Extract of Polygonum cuspidatum attenuates diabetic retinopathy by inhibiting the high-mobility group box-1 (HMGB1) signaling pathway in streptozotocin-induced diabetic rats. Nutrients 8, 140. 10.3390/nu8030140 26950148 PMC4808869

[B58] Song Z. Wei D. Chen Y. Chen L. Bian Y. Shen Y. (2019). Association of astragaloside IV-inhibited autophagy and mineralization in vascular smooth muscle cells with lncRNA H19 and DUSP5-mediated ERK signaling. Toxicol. Appl. Pharmacol. 364, 45–54. 10.1016/j.taap.2018.12.002 30529164

[B59] Stabley J. N. Towler D. A. (2017). Arterial calcification in diabetes mellitus: preclinical models and translational implications. Arterioscler. Thromb. Vasc. Biol. 37, 205–217. 10.1161/ATVBAHA.116.306258 28062508 PMC5480317

[B60] Su X. Wei Y. Cao J. Wu X. Mou D. Luo J. (2018). CCN3 and DLL1 co-regulate osteogenic differentiation of mouse embryonic fibroblasts in a Hey1-dependent manner. Cell Death Dis. 9, 1188. 10.1038/s41419-018-1234-1 30538222 PMC6289993

[B61] Suresh S. McCallum L. Crawford L. J. Lu W. H. Sharpe D. J. Irvine A. E. (2013). The matricellular protein CCN3 regulates NOTCH1 signalling in chronic myeloid leukaemia. J. Pathol. 231, 378–387. 10.1002/path.4246 24308033 PMC4314772

[B62] Tanikawa T. Okada Y. Tanikawa R. Tanaka Y. (2009). Advanced glycation end products induce calcification of vascular smooth muscle cells through RAGE/p38 MAPK. J. Vasc. Res. 46, 572–580. 10.1159/000226225 19571577

[B63] Tian J. Jin D. Bao Q. Ding Q. Zhang H. Gao Z. (2019). Evidence and potential mechanisms of traditional Chinese medicine for the treatment of type 2 diabetes: a systematic review and meta-analysis. Diabetes Obes. Metab. 21, 1801–1816. 10.1111/dom.13760 31050124

[B64] Tu X. Xie C. Wang F. Chen Q. Zuo Z. Zhang Q. (2013). Fructus mume formula in the treatment of type 2 diabetes mellitus: a randomized controlled pilot trial. Evidence-based complementary Altern. Med. eCAM 2013, 787459. 10.1155/2013/787459 PMC3606712 23533521

[B65] Vengrenyuk Y. Carlier S. Xanthos S. Cardoso L. Ganatos P. Virmani R. (2006). A hypothesis for vulnerable plaque rupture due to stress-induced debonding around cellular microcalcifications in thin fibrous caps. Proc. Natl. Acad. Sci. U. S. A. 103, 14678–14683. 10.1073/pnas.0606310103 17003118 PMC1595411

[B66] Wang J. Wang L. Lou G.-H. Zeng H.-R. Hu J. Huang Q.-W. (2019). Coptidis Rhizoma: a comprehensive review of its traditional uses, botany, phytochemistry, pharmacology and toxicology. Pharm. Biol. 57, 193–225. 10.1080/13880209.2019.1577466 30963783 PMC6461078

[B67] Wang T. K. M. Bolland M. J. van Pelt N. C. Horne A. M. Mason B. H. Ames R. W. (2010). Relationships between vascular calcification, calcium metabolism, bone density, and fractures. J. Bone Min. Res. 25, 2777–2785. 10.1002/jbmr.183 20641031

[B68] Wong N. D. Sattar N. (2023). Cardiovascular risk in diabetes mellitus: epidemiology, assessment and prevention. Nat. Rev. Cardiol. 20, 685–695. 10.1038/s41569-023-00877-z 37193856

[B69] Wu S. Luo X. Chen Y. Wang Z. Liu X. Sun N. (2024). Sodium-glucose cotransporter 2 inhibitors attenuate vascular calcification by suppressing endoplasmic reticulum protein thioredoxin domain containing 5 dependent osteogenic reprogramming. Redox Biol. 73, 103183. 10.1016/j.redox.2024.103183 38759418 PMC11127605

[B70] Xu Y. Jiang H. Sun C. Adu-Frimpong M. Deng W. Yu J. (2018). Antioxidant and hepatoprotective effects of purified Rhodiola rosea polysaccharides. Int. J. Biol. Macromol. 117, 167–178. 10.1016/j.ijbiomac.2018.05.168 29802924

[B71] Xue C. Chen K. Gao Z. Bao T. Dong L. Zhao L. (2023). Common mechanisms underlying diabetic vascular complications: focus on the interaction of metabolic disorders, immuno-inflammation, and endothelial dysfunction. Cell Commun. Signal 21, 298. 10.1186/s12964-022-01016-w 37904236 PMC10614351

[B72] Yahagi K. Kolodgie F. D. Lutter C. Mori H. Romero M. E. Finn A. V. (2017). Pathology of human coronary and carotid artery atherosclerosis and vascular calcification in diabetes mellitus. Arterioscler. Thromb. Vasc. Biol. 37, 191–204. 10.1161/ATVBAHA.116.306256 27908890 PMC5269516

[B73] Yang C. Xie Z. Liu H. Wang X. Zhang Z. Du L. (2023). Efficacy and mechanism of Shenqi Compound in inhibiting diabetic vascular calcification. Mol. Med. 29, 168. 10.1186/s10020-023-00767-7 38093172 PMC10720156

[B74] Yao H. Sun Z. Zang G. Zhang L. Hou L. Shao C. (2021). Epidemiological research advances in vascular calcification in diabetes. J. Diabetes Res. 2021, 4461311. 10.1155/2021/4461311 34631895 PMC8500764

[B76] Zhang M.-Y. Zheng S.-Q. (2024). Network pharmacology and molecular dynamics study of the effect of the Astragalus-Coptis drug pair on diabetic kidney disease. World J. Diabetes 15, 1562–1588. 10.4239/wjd.v15.i7.1562 39099827 PMC11292324

[B77] Zhang P. Li Y. Du Y. Li G. Wang L. Zhou F. (2016). Resveratrol ameliorated vascular calcification by regulating sirt-1 and Nrf2. Transplant. Proc. 48, 3378–3386. 10.1016/j.transproceed.2016.10.023 27931585

[B78] Zhang W. Sun Y. Yang Y. Chen Y. (2023). Impaired intracellular calcium homeostasis enhances protein O-GlcNAcylation and promotes vascular calcification and stiffness in diabetes. Redox Biol. 63, 102720. 10.1016/j.redox.2023.102720 37230005 PMC10225928

[B79] Zhang Y. Lin X. Chu Y. Chen X. Du H. Zhang H. (2021). Dapagliflozin: a sodium-glucose cotransporter 2 inhibitor, attenuates angiotensin II-induced cardiac fibrotic remodeling by regulating TGFβ1/Smad signaling. Cardiovasc Diabetol. 20, 121. 10.1186/s12933-021-01312-8 34116674 PMC8196449

[B80] Zhang Y. Wang C. (2011). Nephroblastoma overexpressed (NOV/CCN3) gene: a paired-domainspecific PAX3-FKHR transcription target that promotes survival and motility in alveolar rhabdomyosarcoma cells. Oncogene 30, 3549–3562. 10.1038/onc.2011.69 21423212 PMC3205923

[B81] Zhang Y. Y. Li H. Tu M. X. Mei H Shen X. C. Li J. (2020). The ameliorated effect of Salidroside on phenotype switching of VSMCs by regulating BMP2/Smad1 signaling pathway. J. Southwest Univ. Natl. Sci. Ed. 6, 595–601.

[B82] Zhong H. Li D.-Y. Wang S.-Y. Chen J.-Y. Chen J.-X. Tan X. (2022). Tanshinone IIa attenuates vascular calcification through inhibition of NF-κB and β-catenin signaling pathways. Sheng Li Xue Bao 74, 949–958. 36594383

[B83] Zhu X.-G. Liu G.-Q. Peng Y.-P. Zhang L.-L. Wang X.-J. Chen L.-C. (2024). Exploring the mediating role of calcium homeostasis in the association between diabetes mellitus, glycemic traits, and vascular and valvular calcifications: a comprehensive Mendelian randomization analysis. Diabetol. Metab. Syndr. 16, 136. 10.1186/s13098-024-01383-z 38907296 PMC11193216

