# Supplementary material for: Danlian-Tongmai formula improves diabetic vascular calcification by regulating CCN3/NOTCH signal axis to inhibit inflammatory reaction
Source: Front Pharmacol. 2025 Jan 6;15:1510030. doi: 10.3389/fphar.2024.1510030 (PMC11743396; doi:10.3389/fphar.2024.1510030)
Supplement: Supplementary file 2 [file Table1.docx]

| **Antibody** | **Company** | **Catalog NO.** | **Concentrations** |
| --- | --- | --- | --- |
| RUNX2 | Abcam | ab76956 | WB: 1:1000 |
| BMP2 | Abcam | ab214821 | WB: 1:1000 |
| α-SMA | Proteintech | 14395-1-AP | WB: 1:1000 |
| SM22α | Proteintech | 10493-1-AP | WB: 1:1000 |
| β-actin | Proteintech | 20536-1-AP | WB: 1:5000 |
| CCN3 | Abcam | ab191425 | WB: 1:1000 |
| DLL1 | Proteintech | 28544-1-AP | WB: 1:1000 |
| NOTCH1 | Proteintech | 20687-1-AP | WB: 1:1000 |
| HEY1 | Proteintech | 19929-1-AP | WB: 1:1000 |
| HES1 | Abcam | ab108937 | WB: 1:1000 |
| GAPDH | Proteintech | 10494-1-AP | WB: 1:1000 |

**Table S1. Antibodies**
