# Supplementary material for: Danlian-Tongmai formula improves diabetic vascular calcification by regulating CCN3/NOTCH signal axis to inhibit inflammatory reaction
Source: Front Pharmacol. 2025 Jan 6;15:1510030. doi: 10.3389/fphar.2024.1510030 (PMC11743396; doi:10.3389/fphar.2024.1510030)
Supplement: Supplementary file 4 [file Table5.docx]

| **Protein** | **PDB ID/AlphaFold ID** | **Docking center (x,y,z)** | **Protein pocket size** |
| --- | --- | --- | --- |
| JAGGED1 | 2VJ2 | 17.326, 65.352, 20.148 | 52.236, 131.74, 54.009 |
| HEY1 | 2DB7 | 19.799, 31.334, 3.611 | 28.989, 35.235, 58.127 |
| DLL1 | 4XBM | 18.000, -36.408, 29.261 | 128.287, 167.79, 168.056 |
| NOTCH3 | 4ZLP | 108.526, 18.249, -14.176 | 67.444, 76.722, 83.453 |
| DLL3 | AFQ9NYJ7-F1-model | -13.768, 13.858, -11.109 | 118.748, 72.398, 125.092 |
| DLL4 | 4XL1 | 17.32, -16.635, -16.816 | 59.569, 161.769, 48.015 |
| NOTCH1 | 3ETO | 21.973, -15.26, -10.35 | 67.815, 63.638, 71.087 |
| HES1 | 2MH3 | -4.346, -5.633, -4.193 | 42.439, 57.331, 42.443 |
| CCN3 | 5NB8 | 7.0, 16.3,-2.6 | 88.2, 60.0, 63.8 |

**TableS5 Docking pocket parameters**
