# Supplementary material for: Danlian-Tongmai formula improves diabetic vascular calcification by regulating CCN3/NOTCH signal axis to inhibit inflammatory reaction
Source: Front Pharmacol. 2025 Jan 6;15:1510030. doi: 10.3389/fphar.2024.1510030 (PMC11743396; doi:10.3389/fphar.2024.1510030)
Supplement: Supplementary file 6 [file Table3.docx]

Table S3. Serum biochemical measurements,FBG, bodyweight in

rat by the end of the experiment

| Parameters | CON | DVC | DLTM-L | DLTM-M | DLTM-H | DAPA |
| --- | --- | --- | --- | --- | --- | --- |
| Body weight (g) | 526.09±14.77 | 271.45±20.95^###^ | 243.29±19.80 | 223.18±13.42 | 238.73±22.46 | 238.00±21.83 |
| FBG  (mM) | 6.38±0.14 | 13.88±1.50^##^ | 15.26±1.89 | 13.05±1.97 | 11.25±1.43 | 8.38±0.75^*^ |
| Serum calcium (mM) | 2.45±0.03 | 3.91±0.13^###^ | 3.14±0.17^**^ | 3.25±0.06^**^ | 3.07±0.13^***^ | 3.97±0.08 |
| Serum phosphorus (mM) | 2.39±0.08 | 2.34±0.03 | 2.25±0.11 | 2.29±0.13 | 2.14±0.07 | 2.08±0.03 |

^#^compared with CON group, ^##^*P*＜0.01，^###^*P*＜0.001；^*^compared with DVC group,^*^*P*＜0.05,^**^*P*＜0.001,^***^*P*＜0.001.
