# Supplementary material for: Danlian-Tongmai formula improves diabetic vascular calcification by regulating CCN3/NOTCH signal axis to inhibit inflammatory reaction
Source: Front Pharmacol. 2025 Jan 6;15:1510030. doi: 10.3389/fphar.2024.1510030 (PMC11743396; doi:10.3389/fphar.2024.1510030)
Supplement: Supplementary file 7 [file DataSheet1.docx]

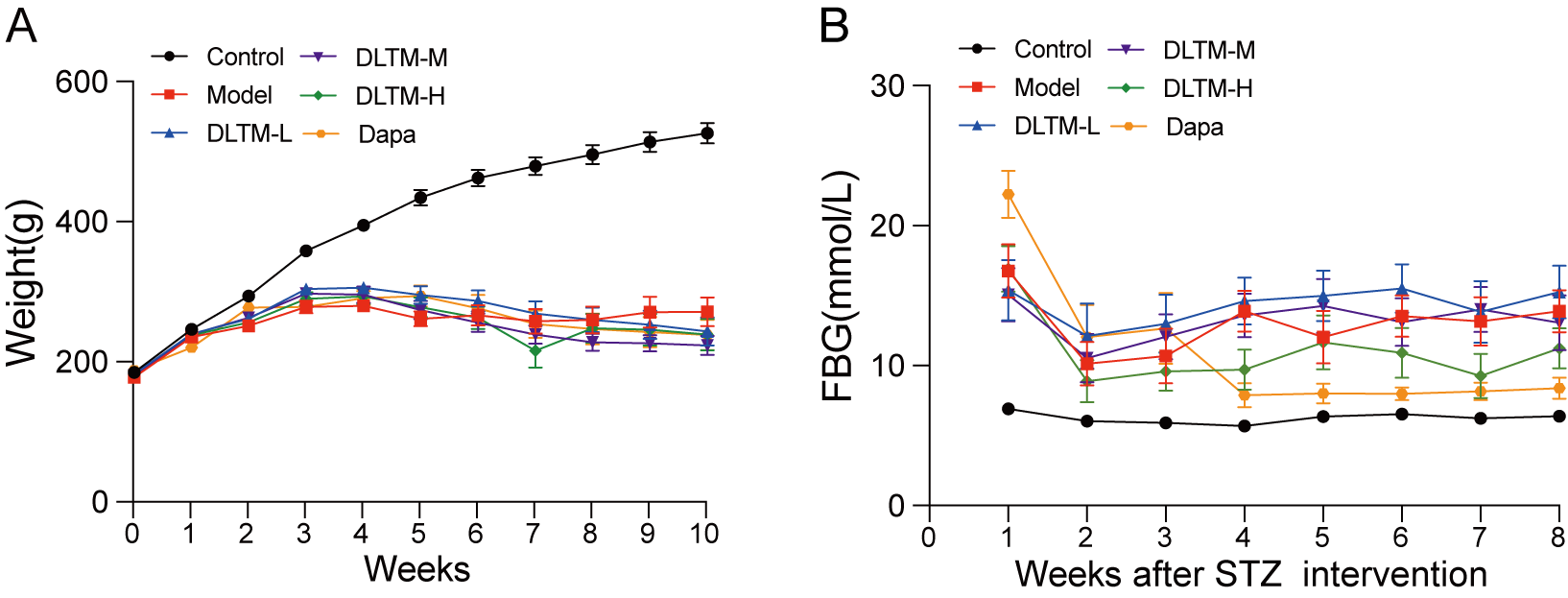


**Figure S1 Changes in body weight and blood glucose levels in each group**

A) Changes of body weight in each group during the experiment (n= 7-11); B) Changes of FBG in rats in each group during the experiment(n = 7-11).

**
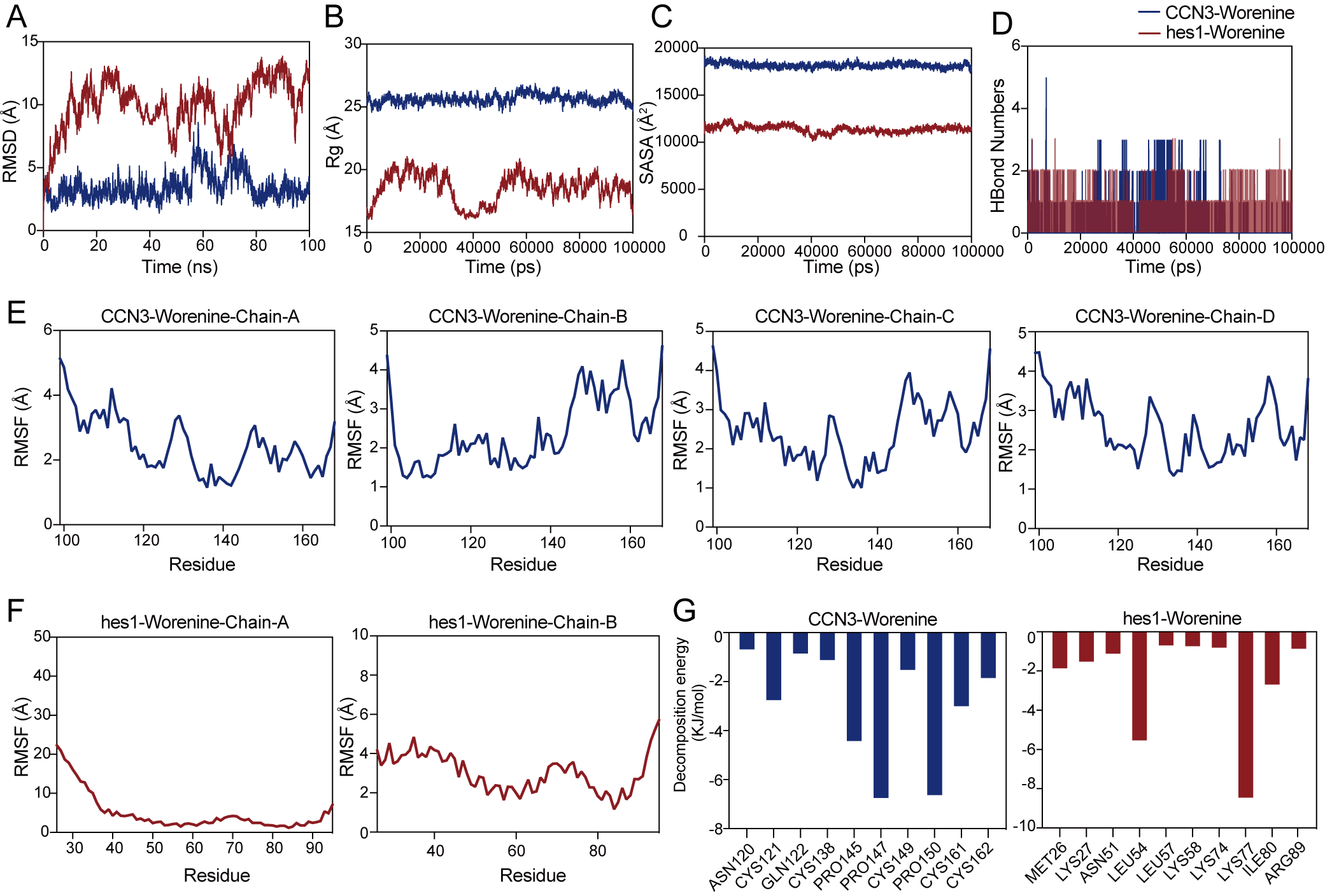
Figure S2 Molecular dynamics simulation of protein-ligand complexes with the best docking effect**

A)RMSD values of the CCN3-Worenine and hes1-Worenine complexes; B) Rg values of CCN3-Worenine and hes1-Worenine complexes;C) SASA of the CCN3-Worenine and hes1-Worenine complex; D) HBonds in CCN3-Worenine and hes1-Worenine complexes during the MD run of 100ns; E) RMSF of backbone atoms in the CCN3-Worenine complex; F) RMSF of backbone atoms in the hes1-Worenine complex; G) Binding Free Energy of CCN3-Worenine and hes1-Worenine complexes. RMSD, represents the root mean square deviation; Rg, radius of gyration; Solvent accessible surface area, SASA; RMSF, root mean square fluctuation.
